# Recent updates on the role of extracellular vesicles in the pathogenesis of allergic asthma

**DOI:** 10.20517/evcna.2021.03

**Published:** 2021-05-12

**Authors:** Ashokkumar Srinivasan, Isaac Kirubakaran Sundar

**Affiliations:** Department of Internal Medicine, Division of Pulmonary, Critical Care and Sleep Medicine, University of Kansas Medical Center, Lawrence, KS 66160, USA.

**Keywords:** Asthma, extracellular vesicles, biomarkers, miRNAs, chronic lung disease

## Abstract

Asthma is a chronic inflammatory disease of the airway diagnosed with different endotypes and phenotypes, characterized by airway obstruction in response to allergens, bacterial/viral infections, or pollutants. Several cell types such as the airway epithelial cells, mesenchymal stem cells and different immune cells including dendritic cells (DCs), T and B cells and mast cells play an essential role during the pathobiology of asthma. Extracellular vesicles (EVs) are membranous nanovesicles produced by every cell type that facilitates intercellular communications. EVs contain heterogeneous cargos that primarily depend on the composition or cell type of origin and they can alter the physiological state of the target cells. EVs encompass a wide variety of proteins including Tetraspanins, MHC classes I and II, co-stimulatory molecules, nucleic acids such as RNA, miRNA, piRNA, circRNA, and lipids like ceramides and sphingolipids. Recent literature indicates that EVs play a pivotal role in the pathophysiology of allergic asthma and may potentially be used as a novel biomarker to determine endotypes and phenotypes in severe asthmatics. Based on the prior reports, we speculate that regulation of EVs biogenesis and release might be under the control of circadian rhythms. Thus, circadian rhythms may influence the composition of the EVs, which alter the microenvironment that results in the induction of an immune-inflammatory response to various environmental insults or allergens such as air pollutants, ozone, diesel exhaust particles, pollens, outdoor molds, environmental tobacco smoke, etc. In this mini-review, we summarize the recent updates on the novel role of EVs in the pathogenesis of asthma, and highlight the link between circadian rhythms and EVs that may be important to identify molecular mechanisms to target during the pathogenesis of chronic inflammatory lung disease such as asthma.

## INTRODUCTION

Asthma is a chronic airway disorder and generally characterized by various symptoms like bronchial hyperreactivity, airway obstruction and inflammation-induced airway remodeling. It was previously described as simple allergic airway inflammation treated with antihistamine and prophylactic agents. Nevertheless, as our understanding of asthma grew deep and wide, it could no longer be considered as a diagnosis term like Parkinson’s or Alzheimer’s. It is an umbrella term that collectively describes the clinical symptoms such as wheezing, breathlessness, chest tightness and cough followed by etiology of asthma. Asthma may be manifested as different endotypes and phenotypes (young atopic, obese middle-aged and elderly asthma). Asthmatics develop clinical symptoms as a result of exposure to different environmental agents that ultimately affect the pathophysiology of the disease^[1,2]^.

Allergens, air pollutants/particulates, ozone, diesel exhaust particles, pollens, microbial infections both bacterial and viral, outdoor molds, environmental tobacco smoke, obesity, exercise, cold air, humidity, genetic predisposition are among the known factors that lead to asthmatic complication either as a result of acute or chronic exposure. Until recently, all asthmatic patients were treated with the same medication. However, this resulted in varying responses to therapies due to the diverse nature of the disease. Therefore, identifying asthma based on the endotype is clinically important to personalize medication for the patients. Asthma is defined as a T2 and non-T2 endotype that includes a range of phenotypes that have similar symptoms, but varying pathophysiology^[3]^. T2 high endotype has three phenotypes [atopic, late-onset, Aspirin exacerbated respiratory disease] and non-T2 endotype has four phenotypes including non-atopic, smokers, obesity-related and elderly asthma. Both innate and adaptive immunity directly regulate asthma phenotypes along with the involvement of immune cells that play a crucial role in the pathogenesis of asthma. Epithelial cells and dendritic cells (DCs) play a major role in determining the type of T helper cells differentiation and activation via cytokine secretion and antigen presentation^[4,5]^. Apart from cytokines, the participation of exosomes in determining the type of immune response elicited against an allergen or environmental insult, is also increasingly getting attention in recent years^[6,7]^. In this review, we discuss the concept of extracellular vesicles (EVs) in determining the endotypes and phenotypes of asthma following exposure to different environmental insults. Understanding the novel role of EVs will help in finding new therapeutic targets for severe asthma that has a very poor clinical outcome.

### Extracellular vesicles

EVs are heterogenous membranous vesicles that cannot replicate (lack of functional nucleus), which includes exosomes, ectosomes and apoptotic bodies. Based on the size, EVs can be classified as exosomes or small EVs (50-200 nm) or Ectosomes (< 200 nm) or apoptotic bodies (200-5000 nm). Exosomes are nano-sized vesicles made up of lipid bilayer membranes enclosing various mixtures of biological molecules, like DNA, RNA, proteins, and lipids^[8,9]^. Exosomes cannot be defined based on their size, as the size range overlaps with the size of microvesicles. Therefore, exosomes are defined based on the mechanism of biogenesis from the host cell. Exosomes are produced by the inward invagination of endosome membrane forming multivesicular bodies (MVB) and ultimately released out of the cell by fusion of MVB with the plasma membrane (exosomes are smaller than MVB themselves) ^[8,9]^. In contrast, ectosomes (microvesicles or microparticles ~100-1000 nm in diameter) are produced by outward budding of the plasma membrane. The last class of EVs are called apoptotic bodies, which are considered as cellular debris and disregarded to have minimal biological function^[9,10]^. Depending on their function, exosome serves as a cargo vehicle for biologically active molecules to distant target tissue have been characterized^[11,12]^. Based on the set of different biomolecules packed into the exosomes, the physiological effect it exerts on the target cells may differ. For example, the exosomes derived from embryonic stem cells are enriched with mRNAs and proteins that are responsible for maintaining pluripotency of the cells and can transfer it to the target cells like hematopoietic stem cells^[13]^. Similarly, microRNAs (miRNAs) that are found in exosomes were shown to be transferred to the target cell and thus functionally silence the target gene^[14,15]^. The exact mechanism behind sorting of specific proteins or molecules inside exosomes are not known. Prior studies have shown that exosomes secreted from the same cells can be packed with different protein profiles thereby proving that exosomes are packed with selective proteins or miRNAs^[16,17]^. Thus, exosomes represent a novel mode of intercellular communication, which may play a major role in many cellular processes, such as immune response, antigen presentation and signal transduction^[18,19]^. Exosome secretion provides cells with an advantage to rapidly release selective molecules and change target cellular response to environmental stimuli or phenotype of the cell. The roles of exosomes on lung pathology are being increasingly described and are being appreciated as immunogenic potentiators especially in the context of allergy^[20]^. Studies to understand the role of EVs in determining the endotypes and phenotypes of asthma (i.e., mediating pro-inflammatory response) following different environmental insults may help in devising novel therapies for asthma.

### Plasma or serum EVs

Plasma or serum samples are easy to obtain from patients with non-invasive procedures that cause minimal pain during sample collection. As miRNAs are known to be highly stable in body fluids, EVs containing miRNAs from serum samples are best suited to study EV biomarkers in asthma. In a recent study, an abundance of miR-122-5p and miR-191-5p were increased in plasma-derived exosomes from patients with asthma without any difference in asthma severity (moderate-severe)^[21]^. They showed levels of miR-122-5p positively correlated with blood eosinophil and neutrophil counts, but not lung function. Similarly, levels of miR-191-5p showed a negative correlation with lung function (FEV1% pred) and percentage of blood lymphocytes. Prior studies showed a strong correlation with blood eosinophils and neutrophils counts/percentages in patients with asthma based on the specific clinical phenotype/endotype of the disease^[22,23]^. Both miR-122-5p and miR-191-5p identified in plasma-derived exosomes from asthmatics (mild-to-moderate or severe eosinophilic asthma) compared to healthy controls may be used as novel biomarkers in asthma after validating these findings using a larger cohort of samples.

Previously, miR-122-5p was known for its proinflammatory property in myocardial infarction and liver diseases^[24,25]^. Prior report showed miR-122-5p expression was increased during lipopolysaccharide (LPS)-induced acute lung injury. Treatment with miR-122-5p inhibitor showed protection against inflammation and injury via modulating DUSP4 (dual specific phosphatase-4) and ERK1/2 signaling pathway^[26]^. Based on the prior report, miR-122-5p may play an important role during lung inflammation/injury and therefore by blocking miR-122-5p expression using a specific inhibitor may offer protection against chronic inflammatory lung disease such as asthma. On the contrary, miR-191-5p promotes inflammation by interacting with key proteins and mRNAs that control Th-2 differentiation and function of APCs^[21]^. Interestingly, miR-191 was also reported to target Bmal1, which is a core clock component that controls circadian rhythms in mouse liver cells^[27]^. Therefore, miR-191 may be a possible key mediator that connects the circadian rhythms-exosome-inflammation axis in asthma. Additional evidence from equine model of severe asthma showed differential expression of several miRNAs (eca-miR-128, eca-miR-744, eca-miR-197, eca-miR-103, eca-miR-107a, eca-miR-30d, eca-miR-140-3p, eca-miR-7, eca-miR-361-3p, eca-miR-148b-3p and eca-miR-215) in serum that regulates airway remodeling and CD4^+^ T cell maturation and differentiation^[28]^. In another human study, miR-125b and miR-126 were found to be increased in exosomes isolated from the serum of asthmatics^[29,30]^. Identifying plasma/serum-derived exosome containing miRNAs may shed light on the hidden molecular and cellular signaling mechanism that controls the immune status in asthma. Therefore, understanding the role of plasma/serum-derived EV miRNA signatures will be important for discovering new circulating biomarkers that can differentiate asthma phenotypes and may help develop targeted therapies in the future.

### Bronchoalveolar lavage fluid EVs

**Figure 1.**
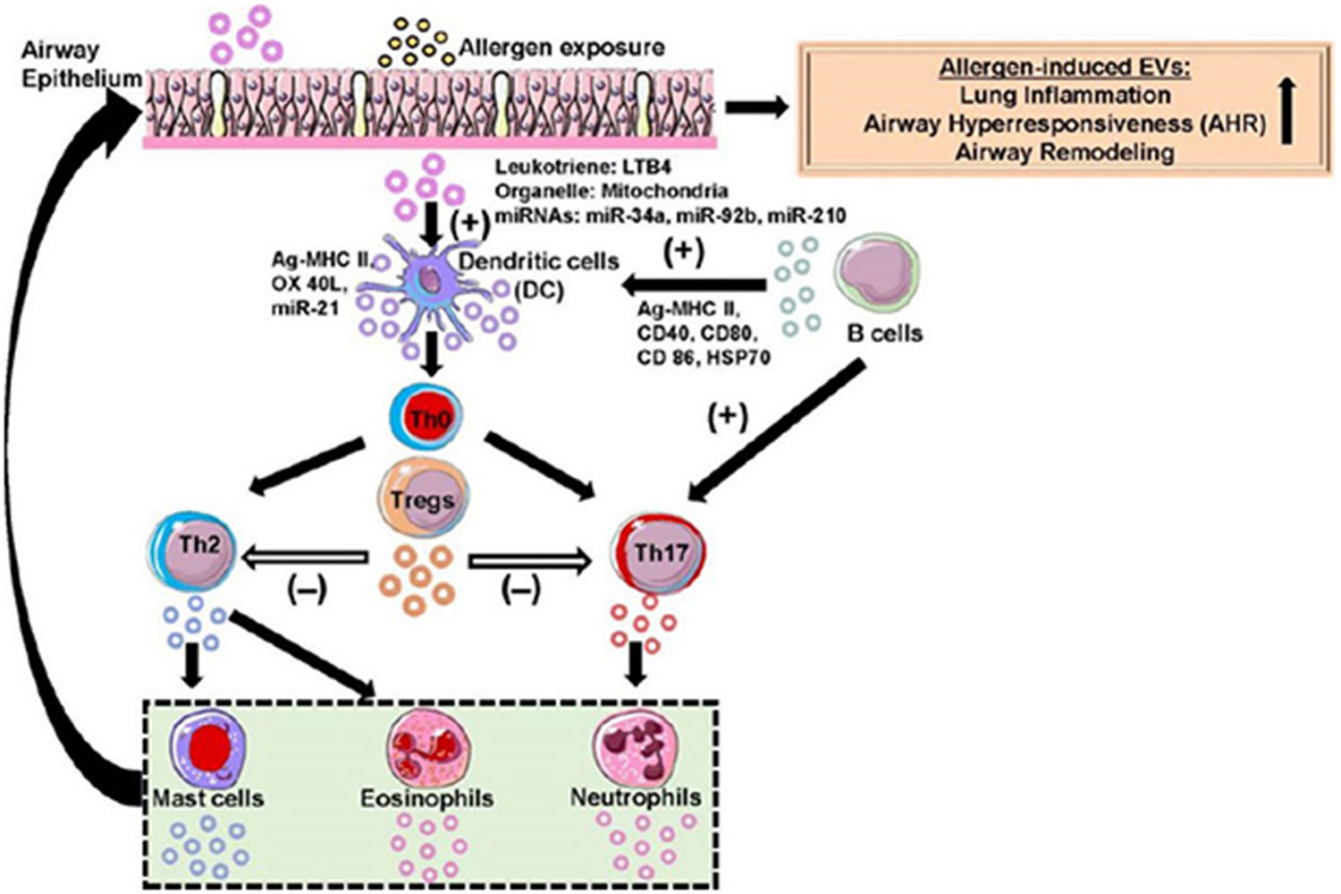
Schematic of extracellular vesicle (EV)-mediated signaling in the lung microenvironment during allergic asthma. A complex interaction occurs between different immune cell-secreted EVs and other target cells (recipient cells) that play a crucial role in the pathophysiology of allergic asthma. Secreted EVs consist of specific biomolecules (e.g., proteins, miRNAs) or organelles (e.g., mitochondria) that cause a phenotypic change in target cells resulting in altered asthmatic lung phenotypes (augmented lung inflammation, airway hyperresponsiveness, and remodeling). EVs have been shown to regulate tissue homeostasis during a normal state and affect target cells leading to the pathobiology of chronic airway disease during a diseased state. EVs released by different immune inflammatory cells (dendritic cells, Th2, Th17, and Tregs, B cells, mast cells, eosinophils, neutrophils, etc.) were represented by an appropriate color of the parent cell and the key biomolecules present in EVs affects the target cells. The directionality of EV-induced changes in recipient cells was indicated using an arrow. EVs that induce cell differentiation or maturation are indicated by a positive symbol (+) and if they inhibit cell maturation or anergy is indicated by a negative symbol (−). This schematic was prepared from SMART (Servier Medical Art), licensed under a Creative Common Attribution 3.0 Generic License. http://smart.servier.com/.

The presence of exosomes in bronchoalveolar lavage (BAL) fluid was characterized for the presence of MHC II and co-stimulatory molecules like CD86 in normal human subjects^[31]^. Though exosomes carry antigen-presenting and co-stimulatory molecules, they do not involve in antigen presentation. Rather they are taken up by professional APCs like dendritic cells, which helps in the activation of naïve T-cells^[32]^. The impact of secreted exosomes in allergic airways has been assessed using animal models. Mice challenged with ovalbumin (OVA) or house dust mite (HDM) show increased secretion of exosomes with different miRNA profiles compared to control mice. Moreover, another similar study showed that treatment with inhibitors of exosome secretion (GW4869) decreased lung inflammation by reducing proliferation and chemotaxis of monocyte^[33,34]^. Exosomes isolated from healthy control subjects and patients with mild intermittent asthma showed 24 differentially expressed miRNAs, which were related to airway inflammation. Let-7 and miRNA-200 family were able to discriminate asthmatics from normal subjects. In particular, miRNAs from miR-200 family that regulate epithelial-mesenchymal transition is downregulated in asthma. Pathway analysis revealed MAPK and JAK-STAT signaling pathway as the most significantly affected by a subset of exosomal miRNAs in asthma^[35]^. A detailed total RNA profiling showed mRNA, miRNAs and other small RNAs (tRNAs, rRNAs, snoRNAs and piRNAs) were significantly altered among healthy controls and severe asthmatics. They showed that several of the miRNAs were downregulated in severe asthmatics (miR-625-3p, miR-202-5p, miR-202-3p, miR-568, and miR-151a-5p) associated with reduced lung function (FEV_1_) and asthma endotype/phenotype that involves neutrophilic (miR-224-5p, miR-581, miR-151a-5p, and miR-9-5p) and eosinophilic infiltration (miR-615-3p, miR-10b-5p and miR-151a-3p)^[36]^ [Table 1 and Figure 1]. Isolation and characterization of BAL fluid exosomes at the molecular level (miRNA profiling) from normal *vs*. asthmatics is an emerging area of research. Studying BAL fluid EVs, their function and their effect on bronchial epithelial cells may open a wide range of possibilities to understand the mechanism of disease and develop new therapeutic strategies.

Exosomes are known to carry many biologically active molecules, but recently it has been discovered that exosomes from BAL fluid contain mitochondria. Especially the exosomes isolated from BAL fluid of asthmatic patients contain more mitochondria compared to healthy volunteers^[37]^. In addition, the study also showed that exosomes from the myeloid-derived regulatory cell (HLA-DR^+^) in BAL could deliver mitochondria to CD4^+^ T cells that induce oxidative stress in T cells to reduce inflammation^[37]^. So far, many studies have delineated the pro-inflammatory property of exosomes, but a study conducted using *BALB/c* mice has demonstrated the tolerogenic (anti-inflammatory) effect of exosomes *in vivo*. When BAL fluid-derived exosomes from mice tolerated to olive pollen (Ole e1) were administered to *BALB/c* mice prior to allergen challenge inhibited IgE response, Th2 cytokine production and airway inflammation by increasing TGF-β in the lung^[38]^. A significant portion of exosomes isolated from BAL fluid of patients sensitized to birch pollen contained mucin 1 indicating that they are mostly derived from bronchial epithelial cells. In addition, BAL fluid-derived epithelial exosomes were packed with leukotrienes synthesizing enzymes (LTA4H, LTC4S, FLAP and 15-LO-1) and are highly capable of synthesizing LTB_4_ leukotrienes compared to antigen-presenting cells (APCs)-derived exosomes. It acts as both chemoattractant for subsets of T lymphocytes and activates lymphocytes as well as DCs. Elevated levels of LTB_4_ from BAL fluid have been reported to increase migration of DC to regional lymph nodes and cause airway hyperresponsiveness *in vivo* in mice ^[39-41]^.

Though most exosomes present in BAL fluid come from airway epithelial cells, a significant proportion of it also comes from resident alveolar macrophage (AM) that is present in the lung cavity. Suppressor of cytokine signaling 3 (SOCS3) plays a significant role in suppressing inflammatory cytokine response and its level were reduced in asthmatics and allergen challenged mice^[42]^. A recent study showed AM-derived EVs were enriched with SOCS3 blocking both STAT3 and STAT6 in human bronchial epithelial cells (BEAS-2B), when challenged with IL-4/IL-13 and HDM. Interestingly, AMs treated with cytokines such as IL-4, IL-33, TSLP and IL-25 contained a very low amount of SOCS3 in both the EVs and cell lysates^[42]^. SOCS3 loaded EVs from AMs may play an important role in the pathogenesis of asthma and synthetic SOCS3 encapsulated liposomes treated in cells and mouse model of allergic asthma showed attenuation of cytokine release and airway inflammation, respectively^[42]^. Hence, synthetic liposomes containing SOCS3 is an emerging therapeutic approach that may be used for the treatment of patients with asthma [Table 1 and Figure 1]. Targeting exosome-mediated LTB_4_ and BLT1 (receptor) pathways may offer alternative therapeutic opportunities to patients with asthma that remain uncontrolled despite intensive corticosteroid treatment.

### Nasal lavage fluid EVs

Lasser *et al*.^[43]^ first reported the existence of EVs in nasal lavage fluid (NLF). They isolated NLF EVs using ultracentrifugation method and characterized by electron microscope, flow cytometer and Western blot analysis for EV- specific markers^[43]^. Like BALF, EVs from the nasal cavity are important for studying inflammation as nose is the first line of defense against inhaled particles such as dust, allergens and air pollutant. Initial observation made by the same group showed that even nasal EVs from healthy subjects were able to promote migration of immune cells like neutrophil, monocyte, and NK cells. Mucin-7, Mucin-5B, Immunoglobulin J chain, polymeric immunoglobulin receptor, filaggrin and hornerin were differentially altered in asthma and asthma with chronic rhinosinusitis condition compared to control^[43]^. Additionally, they also confirmed iNOS activity in isolated NLF EVs from healthy donors^[44]^. Nasal mucous EVs from allergic rhinitis (AR) and healthy controls were characterized for the presence of CD63 and HLA-DR markers. Additionally, they showed 21 miRNAs upregulated and 14 miRNAs downregulated in nasal EVs from AR group compared to healthy controls. Among them, 4 miRNAs (miR-30-5p, miR-199b-3p, miR-28-3p and miR-874) that are involved in B cell receptor signaling pathway were differentially expressed in nasal EVs from AR^[45]^. In a recent study, NLF EVs from asthmatics contained higher amounts of tenascin-C (TN-C) an immunomodulatory extracellular matrix protein in response to human rhinovirus infection. They showed that TLR3, but not TLR7, activation in primary human lung epithelial cells and BEAS-2B cells resulted in TN-C associated EV release. These studies demonstrate TN-C associated EVs are relevant to the biology of viral-induced airway inflammation and exacerbations in asthmatics^[46]^. Compared to BAL fluid, NLFs are easier to collect and process for EV isolation. NLF has a greater advantage over BAL fluid as the nasal cavity forms the first line of defense against allergen and environmental insults. Therefore, early changes in EV release can be detected in nasal exosomes.

### Sputum EVs

Limited studies are available that utilized sputum EVs in asthma. In a study, miRNA signatures from sputum EVs were analyzed in asthmatics. Sputum EVs contained exosome surface markers like CD9, CD63, and HLA-DR as measured by flow cytometry in induced sputum obtained from mild allergic asthma patients before and after allergen challenge. However, there was no significant difference in the surface markers and types of RNA present in sputum exosomes^[47]^. In another study, increased expression of sputum miRNAs (miR-223-3p, miR-142-3p and miR-629-3p) was associated with neutrophilic airway inflammation in mild to moderate and severe asthmatics. All the three miRNAs were upregulated in patients with severe asthma compared to mild asthma, showing a positive correlation with sputum neutrophil percentage and a negative correlation with sputum macrophage percentage^[48]^. The later study reported miRNA transcript levels in sputum sample rather than sputum EVs. Since miRNAs are richly packed in EVs from most biological fluids, this may be correlated with sputum EVs. Future studies exploring miRNA signatures from sputum EVs may be used as a potential diagnostic biomarker to differentiate asthma endotype or phenotypes (e.g., neutrophilic *vs*. eosinophilic asthma).

### Epithelial cell-derived EVs

Epithelial cells are considered an inert physical barriers that protect against inhaled pathogens or allergens. Apart from providing physical and chemical barriers through mucus production, epithelial cells also modulate the immune response to pathogens and allergens evading the mucosal barrier. *In vitro* experiments revealed that exosome exchange between human tracheobronchial epithelial cells (HTBE) and Calu3 cells elevated mucin production (MUC5B and MUC5AC) in HTBE cells, and they are responsible for viscoelastic properties of airway mucus and airway remodeling *in vivo*. Also miRNAs miR-34/449, miR-223 and miR-29 involved in cilia biogenesis were found to be abundant in HTBE exosomes after exosomal exchange^[49]^. Epithelial cells are sensitive to external environmental stimuli such as allergens, bacterial and viral infections, or air pollutants and modulate the innate and adaptive immune response against them. HTBE secretions containing exosome-like vesicles rich in MUC1, MUC4, MUC16, and SNA lectin (α-2,6-linked sialic acid) have been shown to provide innate immune defense against lung pathogens^[49,50]^. Damage to epithelial cells caused by environmental exposure is a common feature in asthma. Ezrin a cytoskeletal protein found on exosomes has been reported to be downregulated in the serum of severe asthmatic patients, with airway epithelial damage caused by IL-13 overexpression^[51,52]^. Tissue factor is another protein present in epithelial cell-derived exosomes involved in airway inflammation and remodeling^[55]^ [Table 1 and Figure 1]. Therefore, proteomic analysis of epithelial cell-derived EVs or BAL fluid exosomes could lead to the identification of a potential new circulating biomarker and novel therapeutic targets for the treatment and management of asthma.

Most of the immunomodulatory effects of epithelial cells were assigned to their cytokine profile, but later studies have shown that exosomes secreted from epithelial cells induced proliferation and chemotaxis of monocytes. On the other hand, inhibition of exosome secretion using an inhibitor (GW4869) is reported to reduce the proliferation of monocytes^[33]^. Depending on the miRNA profile of exosomes secreted by epithelial cells can affect the Th2 polarization and DC maturation response to an allergen or cytokine stimulation. IL-13 treatment in normal human bronchial epithelial cells modulated miRNA profile in secreted exosomes. The study showed miR-34a, miR-92b and miR-210 were significantly decreased in exosomes secreted from epithelial cells^[54]^. All three miRNAs were involved in the regulation of immune cells, e.g., miR-34a is mainly involved in the differentiation of DCs and DC-mediated T cells activation^[54-56]^. Similarly, miR-210 and miR-92b are involved in Th1 and Th17 differentiation and epithelial-to-mesenchymal transition, respectively^[57,58]^ [Table 1 and Figure 1]. Furthermore, future studies on molecular characterization of small RNAs using next-generation sequencing approaches will be possible to predict the difference in endotypes or phenotypes of asthma. Thus, it will enhance our understanding to develop alternative approaches that may be used for treating severe asthmatics. The stability of exosomes isolated from different sources and temperature conditions makes it difficult for further analysis of functional exosomes^[59]^.

### Dendritic cell-derived EVs

DCs play a key role in detecting and directing immune response at the mucosal surface where they constantly sample antigens. This allows them to determine T cell activation and polarization towards Th1, Th2, Treg, or Th17^[60]^. Dendritic cells are well known for their antigen-presenting properties, but they can also secrete exosomes bearing allergens on MHC II. A prior study showed that Fel d1 containing exosomes secreted by monocyte-derived dendritic cells (MDDCs) was able to elicit IL-4 cytokine response in PBMCs isolated from patients, allergic to cat allergen and not in healthy volunteers. In addition, those exosomes can also acquire free allergens and cause the production of IL-4 cytokine in PBMCs of allergic patients^[61]^. They also found that Fel d1 alone caused a similar response in PBMCs, so it is highly doubtful whether the observed effect is due to exosomes or allergen itself. Apart from allergens, exosomes also carry various protein ligand-like OX40L that can specifically help in the proliferation and differentiation of naïve CD4^+^ T cells to Th2 cells^[6]^. Not only the exosomes secreted by dendritic cells, but those secreted by other cells can be taken up by dendritic cells also determine the immunoregulatory effect of DCs. Dendritic cells can also inhibit the differentiation of Th1 and promote Th2 through the STAT3 pathway by secreting exosomes packed with miR-21^[15,62,63]^ [Table 1 and Figure 1]. These studies implicate that allergic airway inflammation and immune response during asthma can be reduced or exaggerated depending on the miRNA cargo present in DC-derived exosomes.

Most of the studies on dendritic cell-derived exosomes were primarily conducted either using BMDCs or MDDCs. The specific dendritic cell subsets like conventional types 1 and 2 dendritic cells (cDC1, cDC2s) and plasmacytoid dendritic cells have a very different role in asthma pathogenesis compared to other types. cDC1s are known for promoting CD8^+^ T cell response, but upon HDM or OVA exposure they induce T cells to differentiate into T reg by producing retinoic acid and activating peroxisome proliferator-activated receptor γ. This helps cDC1s to suppress Th17 and Th2 differentiation upon allergen challenge^[64,65]^. On the other hand, cDC2s are efficient in taking up allergen and effectively migrate towards myeloid lymph nodes to induce Th2 differentiation after allergen challenge in lung airways in animal models^[66-68]^ [Table 1 and Figure 1]. This emphasizes the need for extensive research that will thoroughly isolate and characterize EVs from specific subsets of dendritic cells to understand the mechanism by which allergens dictate the immune response differentially among individuals in a population. This will help in the clinical diagnosis of the endotypes or phenotypes of asthma and direct patients with better alternative therapies, especially among severe asthmatics.

### T and B cell-derived EVs

T cells play a major role in the adaptive immune response during asthma pathogenesis, which are involved in IgE antibody class switching, Th2-related cytokine production, eosinophil recruitment and survival. The mouse model of allergic asthma has established that Th2-mediated immune response is necessary to reproduce features of human asthma^[69]^. APC-dependent allergen priming of CD4^+^ T cells decides the endotype and phenotype of asthma. During this process, T cells form an immune synapse with APCs and secrete exosomes in an antigen-driven unidirectional transfer of exosomal miRNAs toward APCs^[70]^. This mechanism ensures cellular communication between antigen-presenting cells and T cells for effective activation. Prior research demonstrates that T cell activation induces exosomes enriched in surface markers such as TCR-β, CD3ε, CD2, LFA-1 and CXCR4. The exact role of T cell activation-mediated exosome release remains largely unknown, but it has been speculated that they may interact with APCs complement peptide on MHC II to facilitate their function in target cells^[71]^. In another *in vitro* study, T cells activated by IL-2 and CD3/CD28 produced EVs enriched in specific tRNAs that repress the activation of CD4^+^ T cells. It was hypothesized that by utilizing EV biogenesis pathway, T cells get rid of tRNAs that repress its activation as antisense oligonucleotides against the specific tRNA enhance the activation of CD4^+^ T cells^[72]^.

T cells are also responsible for mast cell activation, which has severe consequences in airway inflammation during asthma. Exosomes secreted from activated T cells have been reported to deliver activated Ras GTPase, ZAP70, RASGRP1 and AKT protein to mast cells thereby enhancing mast cell activation in airways^[73,74]^. Th2-mediated inflammation is also promoted by B cell exosomes that are carrying allergen peptides on MHC molecules. There has always been a concern about whether B cells can prime naïve CD4^+^ T cells as they are not professional APCs. In a recent study, B cells have been shown to promote Th2 cytokine response through antigen presentation. Additionally, they showed B cell-restricted MHC II expressing mouse develops Th1 and Th17 immune response when challenged with HDM, but fails to develop Th2 response^[75]^. The antigen-presenting property of B cell-derived exosomes was shown using *in vitro* experiments. The study showed that birch allergen (Bet vi) loaded B cell exosomes induce T cell proliferation and secretion of IL-5 and IL-13 cytokines, which are key drives of airway inflammation and remodeling in asthma^[76]^ [Table 1 and Figure 1]. The immunostimulatory effect of B cell exosomes may play an important role in driving Th2 response *in vivo*. This indicates that the complex interaction and communication between immune cells and APCs dictate specific phenotypic changes during the pathogenesis of asthma.

In a therapeutic approach, Treg cells hold a special place in asthma research as they can effectively ameliorate airway inflammation. Unique lipid signatures from airway exosomes are also reported to promote Th2 and Th17 polarization by modulating membrane fluidity^[77]^. Exosomes secreted by Tregs are referred to as tolerosomes that are quantitatively higher compared to other types of T cells and are regulated by intracellular calcium level and synthesis of sphingolipid^[78,79]^. CircRNA has been isolated and characterized from Treg exosomes, which have been proposed to function as RNA or protein decoy that modulates gene expression^[80]^. The exact mechanism and function of CircRNA remain unclear. This is another area where more research is needed to better understand the role of Treg-derived EVs in the pathogenesis of asthma. Treg exhibits an anti-inflammatory effect via exosomes expressing CD73 on their surface, which induces anti-inflammatory mediators like adenosine^[81]^ [Table 1 and Figure 1].

Repeated exposure of low-dose allergen results in the development of regulatory T cells in the lung, so Tregs may be used as a potential therapeutic target for severe asthma^[82]^. Targeting Treg response in asthma may be an indispensable therapeutic approach, as bacterial exosome (*Pseudomonas aeruginosa*) sensitization in OVA challenged mice show reduced serum levels of IgE, Th2 response and increased Tregs in the lungs^[83]^. Hence, exosomes secreted by Tregs have been implicated to suppress the inflammatory response in different acute and chronic inflammatory diseases. Lack of cell-specific markers to identify the origin of cell-type specific exosome makes it difficult to characterize them in mixed population of EVs from different biofluids. As different types of T cell-specific exosomes carry different sets of miRNAs, they have a wide range of biological functions in asthma. Determining the specific mechanism by which T cell-derived exosomes control other immune cells may be used as a novel biomarker or therapeutic target to treat asthma.

### Eosinophil-derived EVs

One of the prominent features of asthma is eosinophilia and eosinophils are linked with T2 asthma endotype. Eosinophils from asthma patients release more exosomes and they are enriched with eosinophilic proteins like eosinophil peroxidase, major basic protein and eosinophil cationic protein^[84]^. Eosinophil-derived exosomes in healthy and asthmatic conditions share common proteins and are not much different from one another. The only real difference observed was the number of exosomes secreted in asthma patients compared to healthy controls^[85]^. It also induces apoptosis in epithelial cells impeding wound closure and smooth muscle proliferation^[86]^. Eosinophil-derived EVs are unlike any other EVs, there is no report regarding eosinophil-derived EVs or exosomes containing miRNAs or other biomolecules. Limited studies are available that demonstrate the role of eosinophil-derived EVs in asthma^[84,86]^ [Table 1 and Figure 1]. Further studies to characterize the exosomal cargo present in eosinophil-derived EVs such as miRNAs and lipid profiles may lead to a better understanding of how eosinophil-derived EVs alter airway remodeling and hyperresponsiveness during asthma.

### Neutrophil-derived EVs

A small but significant number of people represent asthma with neutrophilia in airways and they have a poor clinical outcome with steroid treatment, unlike eosinophilic asthma. Although neutrophil infiltration can be seen in most asthma endotypes or phenotypes, it is more common in severe asthma^[87]^. Neutrophils play an important role in airway remodeling, inducing allergic inflammation by secreting cytokines, MMP9 and exosomes loaded with LTB4 or its synthesizing enzymes. Neutrophil-derived exosome induces smooth muscle cell proliferation as a result of their uptake by airway smooth muscle (ASM) cells^[88,89]^ [Table 1 and Figure 1]. The proliferation of ASM cells can lead to airway remodeling and airway hyperresponsiveness leading to exacerbation of asthma in severe asthmatics. Additional studies to isolate and characterize neutrophil-derived EVs are needed to better understand their role in the pathogenesis of severe asthma where neutrophilic infiltration plays a crucial role.

### Mast cell-derived EVs

Mast cells (MC) are known for their IgE-mediated effector function in host defense against parasites and allergens. Prior reports have demonstrated the role of mast cells in both innate as well as an adaptive immune response^[90]^. Exosomes from mast cells loaded with antigen (BSA, transferrin and OVA), hsp60 and hsc70 promote DC maturation and antigen presentation of DCs to T cells, thus providing additional evidence that antigen-loaded exosomes facilitate priming of naïve T cell^[91]^. Mast cells treated with IL-4 were able to produce active exosomes that contain immunologically relevant molecules like MHC-II, CD86, LFA-1 and ICAM-1. Therefore, mast cells can activate T and B lymphocytes through exosomes. Antigen-loaded exosomes from mast cells can mount antigen-specific immune response along with DCs^[92]^. Protein and RNA expression analysis revealed many targets that are involved in asthma pathogenesis. Exosome from bone marrow-derived mast cell (BMMC) carrying FcεRI inhibits IgE-mediated MC activation, whereas exosomes secreted by FcεRI activation in MC can cause pro-inflammatory response^[93-95]^. MC exosomes also help in shuffling miRNA between CD34^+^ progenitor cells, controlling maturation processes^[96]^. Airway remodeling is one of the known features in asthma, and epithelial to mesenchymal transition (EMT) plays a significant role in remodeling. MC exosomes were able to initiate a phosphorylation cascade of proteins involved in EMT and upregulate matrix metalloproteases and TGF-β1^[97]^ [Table 1 and Figure 1]. Therefore, identifying novel ways to selectively inhibit specific protein cargo packaged into mast cell-derived EVs might protect against airway inflammation and remodeling in asthma.

### Mesenchymal stem cell-derived EVs

**Figure 2.**
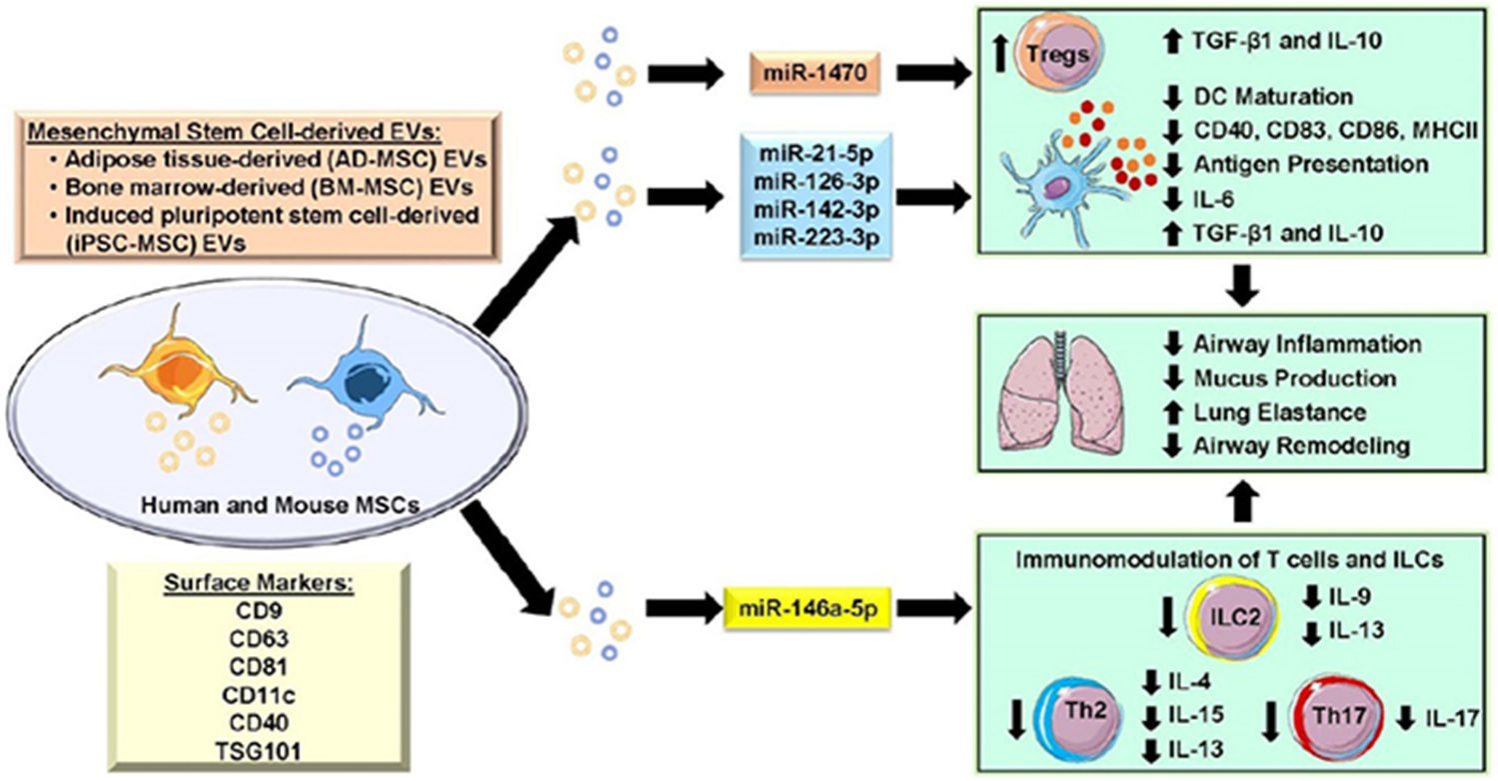
Role of mesenchymal stem cell-derived EVs as novel modulators of lung inflammation and airway remodeling in allergic asthma. EVs isolated from different sources such as bone marrow-MSCs (BM-MSCs), adipose tissues-derived MSCs (AD-MSCs), and induced pluripotent stem cell-derived MSCs (iPSC-MSCs) from human and mouse tissues showed protective response by regulating lung inflammation and remodeling in allergic asthma. MSC-derived EVs contain a wide range of miRNA cargo and other proteins that regulate various aspects of inflammation and immune response *in vitro* and *in vivo*. MSC-derived EVs have been shown to suppress the maturation of dendritic cells (DCs) by downregulating costimulatory molecules, preventing antigen sensitization, and by reducing inflammatory cytokine release in immune cells. Similarly, MSC-derived EVs promote Tregs differentiation, which leads to suppression of Th2 and Th17 immune response in eosinophilic and neutrophilic asthma by producing anti-inflammatory cytokines such as IL-10 and TGF-β1. MSC-derived EVs improve lung function by reducing airway inflammation and remodeling during allergic asthma. This schematic was prepared from SMART (Servier Medical Art), licensed under a Creative Common Attribution 3.0 Generic License. http://smart.servier.com/.

Mesenchymal stem cells (MSCs) are non-hematopoietic stem cells capable of differentiating into osteocytes, adipocytes, chondrocytes and hepatocytes. They are found in adipose tissue, bone marrow and umbilical cord. MSCs have been reported to have an immunomodulatory effect in the microenvironment of tissue by secreting cytokines, immune receptors and EVs/exosomes^[98]^. A recent study demonstrates that exosomes secreted by mouse adipose tissue-derived mesenchymal stromal cells (AD-MSC) decreased interleukin-6 (IL-6), IL-10 and transforming growth factor-β (TGF-β) cytokine release by DCs. It also reduced DCs capacity to induce lymphocyte proliferation and suppressed maturation of bone marrow-derived dendritic cells (BMDCs)^[99]^. Previous studies showed administration of conditioned media or exosomes secreted from bone marrow-derived MSCs or other sources attenuating chronic pulmonary disease. Bone marrow-derived MSC exosomes rather than MSC themselves were effective in reducing IL-10 and TGF-β release by PBMCs from asthmatics^[100]^. Moreover, incubation of MSC exosomes with PBMCs increased Treg population and MSC administration in some cases have been reported to cause vascular occlusion and mal differentiation of MSCs. Using exosomes that have the same or better anti-inflammatory effect could help reduce the undesirable outcomes of the disease^[101]^ [Table 2 and Figure 2].

Studies have also shown that exosomes from human MSCs were effective in reducing Th2 and Th17 cytokines and migration of neutrophil, macrophage and lymphocytes into the lung and BAL fluid in a mouse model of *Aspergillus* hyphal extract-induced allergic airway disease^[102]^. Innate lymphoid cell type 2 (ILC2) plays a very crucial role in airway inflammation and asthma. ILC2 mainly secrets IL-5 a cytokine that acts as a chemoattractant for eosinophils. MSC EVs were able to reduce IL-9^+^ and IL-13^+^ ILC-2, when incubated with PBMCs from patients with allergic rhinitis. In the same study, MSC EVs were able to reduce ILC2 population in the lung, BALF cytokines (IL-5 and IL-13), eosinophil and neutrophil counts in mouse intratracheally administered with recombinant IL-33^[103]^. In an ovalbumin model, exosomes derived from adipose tissue MSCs were able to reduce eosinophils, IL-4, IL-5, Eotaxin and CD4^+^ T cells in the lung and thymus. Additionally, they also showed exosomes from MSCs were able to prevent airway remodeling and improve lung mechanics^[104]^. Anti-inflammatory effects of MSC EVs have been reported in a human study, where the patients affected by graft *vs*. host disease were treated with MSC EVs enriched with anti-inflammatory cytokines like IL-10 and TGF-β1. MSC EVs treatment in patients reduced their steroid dosage significantly^[105]^. This could be tested in patients with asthma as IL-10 and TGF-β1 were reported to play an important role in reducing airway inflammation^[106]^ [Table 2 and Figure 2].

Another report, human bone marrow MSCs-derived EVs reduced maturation markers like CD83, CD38 and CD80 in dendritic cells. It also reduced the production of pro-inflammatory cytokines such as IL-6 and IL-12p70 and increased anti-inflammatory cytokine TGF-β1. The same study showed that miR-21-5p targeted CCR7 receptor degradation in DCs leading to decrease in response to CCL21 and reduced migration into T cell-rich area in the secondary lymphoid organ. This could lead to reduced T cell response to allergen thereby reducing inflammation and possibly eosinophilia or neutrophilia. Another candidate miRNA enriched in MSC EVs is miR-126-3p, which abrogates Th2 response by targeting POU domain class 2 associating factor 1, which indirectly downregulates GATA3 via PU.1 in HDM-induced allergic asthma in BALB/C mice^[107-109]^. On the other hand, miR-142-3p and miR-223-3p were enriched in MSC EVs that augment inflammation by increasing alveolar smooth muscle cell proliferation associated with eosinophilic and neutrophilic infiltration^[110,111]^. Human bone marrow-derived MSC EVs containing miR-1470 show anti-inflammatory properties by increasing FOXP3^+^ Tregs in PBMCs from acute asthmatics. miR-1470 in MSC EVs targets c-Jun mRNA in T cells thereby upregulating P27KIP1 which promotes Treg differentiation^[112]^. Therefore, MSC EVs rather than MSCs themselves may be used as novel therapeutics for the treatment of asthma [Table 2 and Figure 2].

### Circadian rhythms and EVs

Evidence from the literature suggests that EVs could be key regulators of circadian rhythms synchronization between the “donor clock” and “recipient clock”^[113]^. The online EV database such as EVpedia and vesiclepedia provides us with sufficient data that relate to the presence of several proteins/mRNA and miRNAs in EVs^[114-116]^. The proteins such as PER2, PER3, CK1 families, AMPKβ, AMPKγ, GSK-3α, and GSK-3β and other miRNAs such as miR-27b-3p, miR-132, miR-142, miR-192, miR-194, miR-219a, miR-219b, miR-433 and miR-494 were present in EVs. The presence of these miRNAs in EV cargos could regulate clock function in recipient cells. It is noteworthy to determine how EVs participate in cell-to-cell communication between donor *vs*. recipient cells via clock-related molecules and mediate their function locally in tissues and systemically in the circulation^[113]^. Here are a few examples: EVs are responsible for modulating posttranslational modification of clock targets such as GSK3 (phosphorylation), thereby regulating core clock molecules BMAL-CLOCK and CRYs^[117]^. Additional reports suggest that non-coding RNAs in EVs essentially participate in the AMPK-mediated regulation of CRYs^[118]^. Specific miRNAs such as miR-132 and miR-219 have been shown to regulate PERs in a cell-type/tissue-specific manner^[119-121]^. A prior report showed a few of the circulating miRNAs such as miR-142-3p, miR-152 and miR-494 demonstrate diurnal oscillation (peaks at mid-day) and participate in the post-transcriptional regulation of Bmal1^[122]^. In another study, an immune cell type specifically macrophage-derived exosomes containing miR-155 mediates suppressed proliferation and enhanced inflammation in fibroblasts during cardiac injury^[123]^. However, it is unclear if these miRNAs are present in EVs as well or only present as freely circulating miRNAs in serum and how specific miRNAs target the clock-controlled genes (e.g., *bmal1, clock, n1d1, nr1d2, per1-2, cry1-2*, etc.) need to be further explored. A study from mouse model that mimics chronic night shift work revealed changes in plasma EVs, expression of clock genes in target tissues associated with altered metabolic function, and increased permeability of the colonic epithelial cell barrier^[124]^.

The first evidence to demonstrate circadian variation exists in the release of EVs locally in lung tissue/bone marrow cells or systemically in the circulation comes from C57BL/6J mice entrained to a regular light-dark cycle (LD:12/12)^[125]^. EV isolation was performed in lung tissue, whole bone marrow cells and peripheral blood samples collected at 5 different circadian time points (ZT4, ZT8, ZT12, ZT16 and ZT24) revealed time of day-dependent difference in EV concentration^[125]^. Co-culture experiments showed lung-derived EVs alter transcript level of pulmonary specific mRNA and showed a time-dependent change in the uptake of EVs that modulate transcription (increased uptake at ZT20-ZT24)^[125]^. Another report shows the diurnal variation in circulating microvesicles (MVs) (CD41^+^ and Annexin V^+^) to the severity of obstructive sleep apnea (OSA) and the effect of continuous positive airway pressure treatment implicating the role of MVs in the pathobiology of OSA^[126]^. A recent report shows that plasma-derived exosomes from patients with OSA promote endothelial cell senescence (increased p16 and reduced SIRT1 and SIRT6 levels) via oxidative stress-related pathways^[127]^. Additionally, another report using a rat model showed circadian variation in the release of small EV/exosome marker protein. Tumor susceptibility gene 101 (TSG101) displays a circadian pattern to release urinary small EVs (concentration) and that can be used to normalize circadian variation while testing for new EV biomarkers^[128]^. Overall, these data together suggest that circadian rhythms could play a vital role in modulating EV-mediated cell-to-cell communication locally in the lung and systemically in the circulation during normal (healthy) *vs*. diseased state (asthma) that requires thorough investigation in the future. To date there exist no reports that directly link circadian rhythms, EVs, and asthma pathobiology. Furthermore, based on the evidence from the literature there is a strong connection between circadian rhythms and asthma. However, future research will hold accountable to find the missing link and direct role of EVs in circadian rhythms and asthma pathophysiology using novel *in vitro* and *in vivo* approaches.

## CONCLUSIONS AND FUTURE DIRECTIONS

EVs have demonstrated their prime role in causing augmented immune-inflammatory response and airway remodeling during asthma. Additionally, EVs/exosomes from specific cell types such as mesenchymal stem cells and T cells have been shown to mediate protection against chronic inflammatory lung disease. Novel drugs or small molecules that can specifically target EV/exosome trafficking^[129]^ like calpeptin, manumycin A and Y27632, and lipid metabolism such as pantethine, imipramine and GW4869 may help reduce EV-mediated activation of target cells in asthma but will require further investigation. Future studies should investigate how EVs themselves or engineered with specific agents/drugs as biological vehicles can be used to reduce airway inflammation and remodeling in asthma. EVs as novel circulating biomarkers that can be used in the diagnosis, prognosis and therapeutics are expanding further due to the available tools and emerging isolation and characterization technologies^[130]^ such as genomics/transcriptomics, lipidomics, proteomics, metabolomics and high-throughput screening to better understand the role and function of EVs in heath and disease of several different chronic inflammatory lung conditions. Overall, there is a greater need to address the knowledge gap to understand the role of EVs relating to chronic lung diseases. Future studies will provide deeper insights into the complex link between different cellular processes such as circadian rhythms, and associated molecular mechanisms that relate to EV biogenesis, trafficking, cargos (e.g., miRNAs, proteins, lipids, *etc*.), their function and EV-mediated signal transduction that occurs during the pathophysiology of allergic asthma.

## Figures and Tables

**Table 1.** Extracellular vesicles secreted by structural and immune cells and their associated cellular and molecular functions

EV secreted cell-types/ sources	EV isolation and characterization methods	EV molecular signatures	Key findings/major outcomes	Ref.
BAL fluid	Differential ultracentrifugation; Flow cytometry; Immunoelectron microscopy	HLA-DR (MHC II), CD54, CD63, CD86 and MHC I	Human BALF-exosomes are enriched with antigen-presenting molecules like MHC II (HLA-DR), MHC I, CD54 and co-stimulatory molecules like CD86 Enrichment of surface markers in BALF exosomes isolated from different subjects varies widely	[31]
BAL fluid	Differential ultracentrifugation; Western blotting and FACS analysis	MHC I, MHC II and CD9; Presence of SP-B; Ole e 1 in BALF exosome from tolerized mice contains glycosylated and nonglycosylated forms of Ole e 1	BALF-exosomes from mice tolerized to Ole e 1 exosome blocks allergen-specific IgE and IgG_1_ antibodies and reduced Th2 cytokines when pretreated with tolerogenic exosomes, but induces TGF-β	[38]
BAL fluid	Differential ultracentrifugation; FACS analysis	Tetraspanins (CD81, CD63), CD36, HLA-DR (MHC II), MUC1, LTA_4_H, LTC_4_S, FLAP and 15-LO-1	BALF-exosomes from asthmatics showed higher levels of CD81, CD63 and CD36 that correlate with HLA-DR BALF exosomes contained functional proteins that are specific to the leukotrienes pathway	[39]
BAL fluid	Differential ultracentrifugation with modification, FACS analysis; Exosomal miRNA by microarray and validated by RT-PCR	MHC II and CD63 (MHC class I, CD54 and CD86 were not detected); miRNAs: let-7c, let7b, miR-141, miR-200b, let-7d, let-7a, miR-21, miR-27a, let-7e, miR-34c-5p, miR-19b, miR-0022, miR-0024, miR-0026a, miR-0099a, miR-0200c, miR-1972, miR-665, miR-658, miR-483-5p, miR-1268, miR-0203, and miR-0130a	Identified 24 miRNAs in asthmatics vs. healthy control that showed a high correlation with FEV_1_ Downregulated miRNAs: let-7c, let7b, miR-141, miR-200b, let-7d, let-7a, miR-21, miR-27a, let-7e, miR-34c-5p, miR-19b, miR-0022, miR-0024, miR-0026a, miR-0099a, and miR-0200c Upregulated miRNAs: miR-1972, miR-665, miR-658, miR-483-5p, miR-1268, miR-0203, and miR-0130a Validated 8 miRNAs: let-7a, miR-21, miR-658, miR-24, miR-26a, miR-99a, miR-200c, and miR-1268	[35]
BAL fluid	ExoQuick exosome precipitation method; qNano nanoparticle counter; TEM; Affymetrix Gene Chip miRNA 3.0 Array; qRT-PCR Validation	miRNAs: let-7a-5p, miR-702-5p, miR-762, miR-574-3p, miR-574-5p, miR-1827, miR-346, and miR-191-5p	BAL fluid EVs from HDM-treated mice showed an 8.5-fold increase compared to sham control BAL fluid EVs differentially express miRNAs in HDM vs. sham control: let-7a-5p, miR-702-5p, miR-762, miR-574-3p, miR-574-5p, miR-1827, miR-346, and miR-191-5p; Validated miRNAs in EVs: (upregulated: miR-346, miR-1827 and miR-574-5p) GW4869 treatment reduced EV release and EV miRNAs: miR-1827, miR-574-5p and miR-346 and HDM-induced allergic airway inflammation (differential cell counts and associated Th2 cytokines)	[34]
BAL fluid	Differential ultracentrifugation; NanoSight NS300; TEM; Image Stream analysis; Flow cytometry; SWATH lipidomic analysis	HLA-DR^+^ and CD54^+^ (ICAM-1); CD9^+^CD63^+^CD81^+^TSG101^+^ Phosphatidylglycerol 34:2, ceramide-phosphate 28:0, and ceramide 34:2	Increased expression HLA-DR^+^ and CD54^+^ in BAL fluid-derived EVs from asthmatics compared to healthy control Lipidomic analysis reveals reduced levels of phosphatidylglycerol, ceramide-phosphates, and ceramides in exosomes from asthmatics compared to healthy control Sphingomyelin 34:1 was abundant in secondhand-smoke-exposed asthmatics compared to healthy controls	[78]
BAL fluid	Differential ultracentrifugation; NanoSight NS300; TEM; Image Stream analysis; Flow cytometry; CryoEM	CD63, HLA-DR, mitochondrial DNA (mt-DNA) and mitochondria	EVs isolated from BAL fluid of asthmatics and myeloid-derived regulatory cells (MDRC)-derived exosomes contain mitochondria (MitoT-Green^+^ and mitochondrial DNA) MDRC-derived exosomes contain polarized mitochondria that when transferred to T cells result in the formation of a mitochondrial network.	[37]
BAL fluid	Exo-Mir kit for nanovesicle RNA from 20 ml BAL fluid; Small RNA-seq analysis and Validation by qPCR	miRNAs: miR-625-3p, miR-202-5p, miR-202-3p, miR-568, miR-151a-5p, miR-615-3p, miR-10b-5p, miR-151a-3p, miR-224-5p, miR-581, and miR-9-5p	Specific human miRNAs were downregulated in severe asthmatics correlated with FEV_1_ and immune-inflammatory phenotypes (eosinophilic *vs*. neutrophilic inflammation or Atopy)	[36]
NHBE and BAL fluid 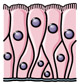	Differential ultracentrifugation; TEM; Immunoblotting	Tissue factor (TF), EGFR, and Annexin V	Compression stress-induced TF-containing exosomes in differentiated normal human bronchial epithelial cells (NHBE) TF levels were elevated in human BALF-derived exosomes from asthmatics	[53]
BAL fluid/Epithelial cells 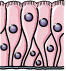	Differential ultracentrifugation; Bead-based assay; ELISA; TEM; Flow cytometry	MHC II, HSP70, CD63	IL-13 treatment induced exosome secretion in BEAS-2B cells to facilitate monocyte proliferation and chemotaxis GW4869-mediated reduction in exosomes reduced monocyte/macrophage infiltration in the lungs of OVA-induced mouse model	[33]
Epithelial cells 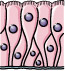	Differential ultracentrifugation with modifications; Nano-LC-ESI MS/MS analysis; Immuno EM and flow cytometry	MUC1, MUC4, MUC16, SNA lectin (α-2,6-linked sialic acid), keratan sulfate (5D4), CD59, CD63, MHC class I and II	Mucin and sialic acid lectin enriched exosome confer innate immune defense against viral infection	[50]
Epithelial cells 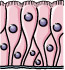	Diffrential ultracentrifugation with modifications; NanoSight NS300; TEM; MS analysis; HTG EdgeSeq miRNA	Mucins (MUC3B, MUC13, MUC5AC and MUC5B) and miRNAs (miR-34/449, miR-223 and miR-29)	MUC4 is unique to human tracheobronchial epithelial cells (HTBE)-derived-exosomes and MUC13 and MUC3B are unique to Calu3-exosomes that confer innate defense and contribute to viscoelastic properties to airways Calu-3 treated exosomes showed increased expression levels of miR-3180 and miR-3180-3p Increased expression of these miRNA targets (miR-18a-5p, miR-19a-3p, miR-141-3p, miR-200a-3p, miR-200c-3p, miR-29a-3p, and miR-29b-1-5p) were observed in Calu-3 exosomes and subsequently when Calu-3 exosomes were treated in HTBE cells in their exosomes	[49]
NHBE 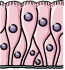	qEV columns (size-exclusion chromatography); ExoQuick-TC reagent; PMX 110 scanning ZetaView; TEM, SeramiR miRNA prolifing; RT-qPCR	CD63, CD9 and CD81 miRNAs downregulated in IL-13 treated NHBEs: miR-210, miR-125a-5p, miR-34a, miR-92b, miR-210	IL-13 treatment alters the miRNA signatures (majority of the miRNAs were downregulated: e.g., miR-210, miR-125a-5p, miR-34a, miR-92b, miR-210, etc.) in apical and basal epithelial cell-derived EVs (both early *vs*. late) are involved in Th2 differentiation and DC maturation Nasal lavage sEVs showed decreased expression of miR-34a, miR-92b and miR-210 correlated with airway obstruction in children	[54]
Fibroblast 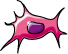	Differential ultracentrifugation with modification; TEM; flow cytometry	CD63	Bronchial fibroblast-derived exosomes from severe asthmatics compared to normal bronchial fibroblasts (healthy control) showed lower levels of cytokine TGF-β2 and control epithelial proliferation and repair	[7]
Dendritic cells 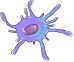	Differential ultracentrifugation with modification; Human microRNA microarray from Agilent; Flow cytometry	miRNAs: miR-335 miR-760, miR-632, miR-654-5p, miR-671-5p, miR-92a, miR-32, miR-101 and miR-21	Exosomal miR-335 (from primary DCs) is transferred from T cells to APC in an antigen-specific manner Transferred miRNA regulates gene expression of APC	[70]
Dendritic cells 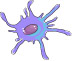	Differential ultracentrifugation; NanoSight LM10; Immuno-EM; Flow cytometry	MDDCs exosomes and exosomes rFeld1 contains HLA-DR, CD63 and CD81	Exosomes from monocyte-derived dendritic cells (MDDC) carry rFel d1 (cat allergen) and induce IL-4 production in PBMCs from cat-allergenic individuals Demonstrated distribution of aeroallergens via exosomes derived from DCs	[61]
Dendritic cells 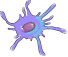	Differential ultracentrifugation; ELISA; Flow cytometry	CD63 and OX40L	TSLP-activated DCs released exosomes that are enriched in OX40L promote Th2 differentiation	[6]
T cells 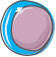	Differential ultracentrifugation; flow cytometry	Microvesicles from Jurkat T cells and human CD4^+^ T cells and CD8^+^ T cells clone contain CD3ε/ζ complex	Microvesicles from Jurkat cells and T lymphoblasts expressed CD3ε, TCR and CD63 Microvesicles from CD3-activated Jurkat cells and lymphoblasts showed expression of CD2, CD18, CXCR4, and MHC I and to a lesser extent MHC II	[71]
T cells 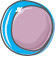	Differential ultracentrifugation; Immunoelectron microscopy; FACS analysis	Microparticles from resting and activated T cells contain LFAα/CD11α	Microparticles-derived from activated T cells induces mast cell degranulation (increase in the release of β-hexosaminidase) Microparticles from activated T cells induce cytokine release (IL-8 and oncostatin M) and activate ERK phosphorylation in human mast cells	[74]
T cells 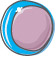	Differential ultracentrifugation; Human microRNA microarray (Agilent); Flow cytometry	miRNAs are abundant in Jurkat-derived J77 T cell exosomes (miR-760, miR-632, miR-654-5p and miR-671-5p).	Differentially expressed miRNAs in T cells *vs*. T cell-derived exosomes: (upregulated: miR-760, miR-632, miR-654-5p, miR-671-5p) and (downregulated: miR-32, miR-101, miR-21)	[70]
T cells 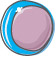	Differential ultracentrifugation; flow cytometry; mass spectrometry analysis	Proteins: RAS, ZAP70, RASGRP1, AKT, CD63 and CD81	Jurkat T cell-derived exosomes transfer proteins of RAS/MAPK signaling pathways (RAS, ZAP70, RASGRP1 and AKT) to mast cells and activate ERK phosphorylation *in vitro*	[73]
Tregs 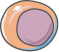	Differential ultracentrifugation; TEM; Flow cytometry	CD81, CD63 and CD73	TCR activation leads to the secretion of exosomes from CD4^+^ CD25^+^ Tregs enriched in CD73 mediates immune suppression via adenosine production	[81]
B cells 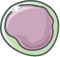	Differential ultracentrifugation; Flow cytometry	MHC classes I and II, CD40, CD 54, CD63, CD80, CD81, CD86 and CD19	B cell-derived exosomes can bind peptides derived from Bet v 1 (Birch peptides) B cell-derived exosomes loaded with Bet v 1 peptides can induce T cell proliferation as well as increase cytokine production in allergen-specific T cells	[76]
B cells 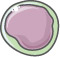	Differential ultracentrifugation; Human microRNA microarray from Agilent; Flow cytometry	miRNAs: miR-760, miR-632, miR-654-5p and miR-671-5p and miR-32	miRNAs abundant in Raji B cell exosomes (miR-760, miR-632, miR-654-5p and miR-671-5p) Differentially expressed miRNAs in B cells vs. B cell-derived exosomes: miR-760, miR-632, miR-654-5p and miR-671-5p (upregulated) and miR-32 (downregulated)	[70]
Eosinophils 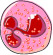	Differential ultracentrifugation; NanoSight LM10; TEM, flow cytometry, Immunoblot analysis	Eosinophil peroxidase (EPO), Major basic protein (MBP), Eosinophil cationic protein (ECP)	Eosinophil-derived exosomes were enriched with eosinophil granule proteins such as EPO, MBP and ECP without any significant difference among asthmatics *vs*. healthy controls	[85]
Eosinophils 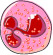	Differential ultracentrifugation; NanoSight LM10; Exosome proteins by RP-LC-MS/MS	EPO, MBP, ECP and periostin	High number of eosinophil-derived exosomes with basic proteins (EPO, MBP, and ECP) were detected in eosinophils from patients with asthma compared to healthy controls Eosinophil-derived exosomes produce ROS and NO	[84]
Eosinophils 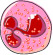	Differential ultracentrifugation	-	Eosinophil-derived exosomes from asthmatics delay wound healing, induce apoptosis and cytokine secretion (TNF, CCL26 and POSTN) in SAEC via PI3K/AKT and JAK-STAT signaling Eosinophil-derived exosomes from asthmatics increased expression of both angiogenesis and fibrosis markers (*CCR3* and *VEGFA*) in bronchial smooth muscle cells	[86]
Neutrophils 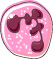	Differential ultracentrifugation and size-exclusion chromatography; electron microscopy; NICOMP 30 device; Nano-LC-MS/MS	Phosphatidylcholine-sterol acyltransferase, Tenascin, Thrombospondin-1, Annexin A7, Neurogenic locus notch homolog protein 2, Lactotransferrin, Integrin-linked protein kinase, Protein S100A9, Fibrinogen A-α chain, Serpin peptidase inhibitor, clade B, member 1, Lipocalin 2, α-1-acid glycoprotein 2, Complement C3, Profilin-1, Triosephosphate isomerase, Integrin-β2	Identified proteins were differentially expressed between unstimulated *vs*. LPS-stimulated neutrophil-derived exosomes LPS-stimulated neutrophil-derived exosomes showed an increase in smooth muscle proliferation implicating their role in airway remodeling	[88]
Mast cells 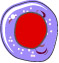	Differential ultracentrifugation	Hsp60 and hsc70	MC-derived exosomes induce DC maturation *in vitro* BMMC exosomes show selective enrichment of hsp60 and hsc70 MC-derived exosomes induce DCs to become efficient APCs	[91]
Mast cells 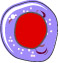	Differential ultracentrifugation; FACS analysis; Immuno-EM; ELISA and MALDI-TOF-MS	Hsp60, hsc70, MHC II, CD86, CD40, CD40L, LFA-1 and ICAM-1	BMMC-derived exosome enriched in these surface markers (MHC II, CD86, CD40, CD40L, LFA-1 and ICAM-1) induces dendritic cells BMMC-derived exosomes loaded with BSA when injected in mice stimulate B and T lymphocytes	[92]
Mast cells 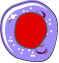	Differential ultracentrifugation; TEM, Immunoblotting; FACS analysis; confocal microscopy	BMMC exosomes contained FcεRI	BMMC-derived exosomes can bind to free IgE via FcεRI BMMC-derived exosomes showed reduced airway inflammation, AHR in the OVA-induced allergic asthma	[94]
Mast cells 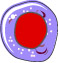	Differential ultracentrifugation	-	MC-derived EVs upregulate epithelial-mesenchymal transition markers (*TGFB1, TWIST1, MMP9* and *BMP7*) at the transcript level in epithelial cells MC-derived EVs induced a mesenchymal-like phenotype and phosphorylation of several protein targets in epithelial cells	[97]

AHR: Airway hyperresponsiveness; BMMC: bone marrow-derived mast cell.

**Table 2.** Mesenchymal stem cell (MSC)-derived EVs as novel therapeutics for allergic asthma

EV secreted cell- types	EV isolation and characterization methods	EV molecular signatures	Key findings/major outcomes	Ref.
MSCs 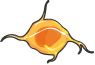	Differential Ultracentrifugation; NanoSight NS300; TEM	-	Administration of either mMSC or hMSC-derived EVs differentially reduced airway hyperresponsiveness, lung inflammation, and Th1, Th2 and Th17 cytokines in *Aspergillus* hyphal extract-induced allergen model	[102]
Human AD-MSCs 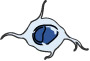	Differential Ultracentrifugation; Zetasizer Nano ZS90 system; SEM	-	Human adipose tissue-derived MSC (AD-MSC)-derived EVs reduced eosinophil counts in the lung tissue and BALF associated with reduced airway inflammation and remodeling in OVA-induced allergic asthma	[104]
Mouse AD-MSCs 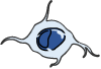	Bead-based isolation method; Dynamic light scattering (DLS); SEM; TEM	CD86, CD40, CD11c	Murine AD- MSC-derived exosomes reduced LPS-induced IL-6, IL-10, and TGF-β cytokine release in DCs Lymphocyte proliferation was reduced in DCs treated with MSC-derived exosomes MSC-derived exosomes suppressed the maturation of BMDCs that are key players in modulating DC-induced immune response	[99]
Human BM-MSC 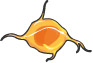	Differential Ultracentrifugation; TEM	CD9, CD81	MSC-derived exosomes promote Treg proliferation in PBMCs from asthmatics and healthy controls MSC-derived exosomes significantly increased anti-inflammatory cytokines IL-10 and TGF-β1 production in supernatant from PBMCs compared to control MSC exosomes incubated with PBMCs induced Tregs differentiation	[101]
Human iPSC MSC 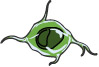	Differential ultracentrifugation; Anion-exchange chromatography TEM	CD9, CD63, CD81	Human MSC-derived small EV (sEV) blocked ILC2 function in PBMCs from patients with allergic rhinitis in response to IL-2/IL-25/IL-33 Additionally, MSC-sEV reduced IL-9 and IL-13 release in the supernatants of sorted ILC2s following IL-2/IL-25/IL-33 treatment Systemic administration of MSC-sEV attenuated ILC2-dominant allergic airway inflammation caused by IL-33 exposed mice (reduced total cells, eosinophils and neutrophils and IL-5 and IL-13 release in BALF, mucus production including reduced ILC2s in the lung) RNA-seq analysis of MSC-sEV revealed miR-146a-5p transcript level significantly upregulated Finally, miR-146a-5p present in MSC-sEV role in modulating the effects of ILC2 *in vitro* in PBMCs and *in vivo* in mice were proven using miR-146a-5p inhibitor and mimics	[103]
Human BM-MSC 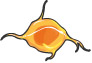	Differential Ultracentrifugation; TEM; N TA	CD63, CD9, CD81 miR-21-5p, miR-142-3p, miR-126-3p.	Labeled MSC-EVs preferentially targets DCs when co-cultured with T cells as shown by colocalization of MSC-EVs and DCs MSC-EVs treated immature DCs reduced up take of FITC dextran suggesting their role in inhibiting DC maturation and antigen presentation MSC-EVs impairs DC migration to lymph nodes by reducing CCR7 expression MSC-EVs contained miRNAs (miR-21-5p, miR-142-3p, and miR-126-3p) which exerts an effect on DC maturation and function	[107]

## References

[1] Chung KF, Wenzel SE, Brozek JL, International ERS/ATS guidelines on definition, evaluation and treatment of severe asthma. Eur Respir J 2014;43:343–73. 24337046 10.1183/09031936.00202013

[2] Kaur R, Chupp G. Phenotypes and endotypes of adult asthma: moving toward precision medicine. J Allergy Clin Immunol 2019; 144:1–12. 31277742 10.1016/j.jaci.2019.05.031

[3] Kuruvilla ME, Lee FE, Lee GB. Understanding asthma phenotypes, endotypes, and mechanisms of disease. Clin Rev Allergy Immunol 2019;56:219–33. 30206782 10.1007/s12016-018-8712-1 PMC6411459

[4] Woodruff PG. Subtypes of asthma defined by epithelial cell expression of messenger RNA and microRNA. Ann Am Thorac Soc 2013;10:S186–S9. 24313771 10.1513/AnnalsATS.201303-070AW PMC3960987

[5] Vroman H, Hendriks RW, Kool M. Dendritic cell subsets in asthma: impaired tolerance or exaggerated inflammation? Front Immunol 2017;8:941. 28848549 10.3389/fimmu.2017.00941 PMC5552666

[6] Huang L, Zhang X, Wang M, Exosomes from thymic stromal lymphopoietin-activated dendritic cells promote Th2 differentiation through the OX40 ligand. Pathobiology 2019;86:111–7. 30408778 10.1159/000493013

[7] Haj-Salem I, Plante S, Gounni AS, Rouabhia M, Chakir J. Fibroblast-derived exosomes promote epithelial cell proliferation through TGF-β2 signalling pathway in severe asthma. Allergy 2018;73:178–86. 28649804 10.1111/all.13234

[8] Bobrie A, Colombo M, Raposo G, Théry C. Exosome secretion: molecular mechanisms and roles in immune responses. Traffic 2011;12:1659–68. 21645191 10.1111/j.1600-0854.2011.01225.x

[9] Théry C, Witwer KW, Aikawa E, Minimal information for studies of extracellular vesicles 2018 (MISEV2018): a position statement of the International Society for Extracellular Vesicles and update of the MISEV2014 guidelines. J Extracell Vesicles 2018;7:1535750. 30637094 10.1080/20013078.2018.1535750 PMC6322352

[10] Kakarla R, Hur J, Kim YJ, Kim J, Chwae Y-J. Apoptotic cell-derived exosomes: messages from dying cells. Exp Mol Med 2020;52:1–6. 31915368 10.1038/s12276-019-0362-8 PMC7000698

[11] Simpson RJ, Lim JW, Moritz RL, Mathivanan S. Exosomes: proteomic insights and diagnostic potential. Expert Rev Proteomics 2009;6:267–83. 19489699 10.1586/epr.09.17

[12] Waldenström A, Gennebäck N, Hellman U, Ronquist G. Cardiomyocyte microvesicles contain DNA/RNA and convey biological messages to target cells. PLoS One 2012;7:e34653. 22506041 10.1371/journal.pone.0034653 PMC3323564

[13] Ratajczak J, Miekus K, Kucia M, Zhang J, Reca R, Embryonic stem cell-derived microvesicles reprogram hematopoietic progenitors: evidence for horizontal transfer of mRNA and protein delivery. Leukemia 2006;20:847–56. 16453000 10.1038/sj.leu.2404132

[14] Valadi H, Ekström K, Bossios A, Sjöstrand M, Lee JJ, Lötvall JO. Exosome-mediated transfer of mRNAs and microRNAs is a novel mechanism of genetic exchange between cells. Nat Cell Biol 2007;9:654–9. 17486113 10.1038/ncb1596

[15] Montecalvo A, Larregina AT, Shufesky WJ, Mechanism of transfer of functional microRNAs between mouse dendritic cells via exosomes. Blood 2012;119:756–66. 22031862 10.1182/blood-2011-02-338004 PMC3265200

[16] Tauro BJ, Greening DW, Mathias RA, Mathivanan S, Ji H, Simpson RJ. Two distinct populations of exosomes are released from LIM1863 colon carcinoma cell-derived organoids. Mol Cell Proteomics 2013;12:587–98. 23230278 10.1074/mcp.M112.021303 PMC3591653

[17] Palma J, Yaddanapudi SC, Pigati L, MicroRNAs are exported from malignant cells in customized particles. Nucleic Acids Res 2012;40:9125–38. 22772984 10.1093/nar/gks656 PMC3467054

[18] Barros FM, Carneiro F, Machado JC, Melo SA. Exosomes and immune response in cancer: friends or foes? Front Immunol 2018;9:730. 29696022 10.3389/fimmu.2018.00730 PMC5904196

[19] Kawahara H, Hanayama R. The role of exosomes/extracellular vesicles in neural signal transduction. Biol Pharm Bull 2018;41:1119–25. 30068858 10.1248/bpb.b18-00167

[20] Admyre C, Telemo E, Almqvist N, Exosomes - nanovesicles with possible roles in allergic inflammation. Allergy 2008;63:404–8. 18315728 10.1111/j.1398-9995.2007.01600.x

[21] Bahmer T, Krauss-Etschmann S, Buschmann D, RNA-seq-based profiling of extracellular vesicles in plasma reveals a potential role of miR-122-5p in asthma. Allergy 2021;76:366–71. 32627209 10.1111/all.14486 PMC7818394

[22] Zhang XY, Simpson JL, Powell H, Full blood count parameters for the detection of asthma inflammatory phenotypes. Clinical & experimental allergy 2014;44:1137–45. 24849076 10.1111/cea.12345

[23] Gungen AC, Aydemir Y. The correlation between asthma disease and neutrophil to lymphocyte ratio. Res J Allergy Immunol 2017; 1:1–4.

[24] Cortez-Dias N, Costa MC, Carrilho-Ferreira P, Circulating miR-122-5p/miR-133b ratio is a specific early prognostic biomarker in acute myocardial infarction. Circulation journal 2016;80:2183–91. 27593229 10.1253/circj.CJ-16-0568

[25] Jin Y, Wong YS, Goh BKP, Circulating microRNAs as potential diagnostic and prognostic biomarkers in hepatocellular carcinoma. Scientific Reports 2019;9. 10.1038/s41598-019-46872-8 PMC6639394 31320713

[26] Lu Z, Feng H, Shen X, MiR-122-5p protects against acute lung injury via regulation of DUSP4/ERK signaling in pulmonary microvascular endothelial cells. Life Sci 2020;256:117851. 32470454 10.1016/j.lfs.2020.117851

[27] Na YJ, Sung JH, Lee SC, Comprehensive analysis of microRNA-mRNA co-expression in circadian rhythm. Exp Mol Med 2009;41:638–47. 19478556 10.3858/emm.2009.41.9.070 PMC2753657

[28] Pacholewska A, Kraft MF, Gerber V, Jagannathan V. Differential expression of serum microRNAs supports CD4^+^ T cell differentiation into Th2/Th17 cells in severe equine asthma. Genes (Basel) 2017;8:383. 10.3390/genes8120383 PMC5748701 29231896

[29] Zhao M, Juanjuan L, Weijia F, Expression levels of microRNA-125b in serum exosomes of patients with asthma of different severity and its diagnostic significance. Curr Drug Metab 2019;20:781–4. 31631818 10.2174/1389200220666191021100001

[30] Zhao M, Li YP, Geng XR, Expression level of miRNA-126 in serum exosomes of allergic asthma patients and lung tissues of asthmatic mice. Curr Drug Metab 2019;20: 799–803. 31608839 10.2174/1389200220666191011114452

[31] Admyre C, Grunewald J, Thyberg J, Exosomes with major histocompatibility complex class II and co-stimulatory molecules are present in human BAL fluid. Eur Respir J 2003;22:578–83. 14582906 10.1183/09031936.03.00041703

[32] Mallegol J, Van Niel G, Lebreton C, T84-intestinal epithelial exosomes bear MHC class II/peptide complexes potentiating antigen presentation by dendritic cells. Gastroenterology 2007; 132:1866–76. 17484880 10.1053/j.gastro.2007.02.043

[33] Kulshreshtha A, Ahmad T, Agrawal A, Ghosh B. Proinflammatory role of epithelial cell–derived exosomes in allergic airway inflammation. J Allergy Clin Immunol 2013;131:1194–203, 1203.e1-14. 23414598 10.1016/j.jaci.2012.12.1565

[34] Gon Y, Maruoka S, Inoue T, Selective release of miRNAs via extracellular vesicles is associated with house-dust mite allergen-induced airway inflammation. Clin Exp Allergy 2017;47:1586–98. 28859242 10.1111/cea.13016

[35] Levänen B, Bhakta NR, Torregrosa Paredes P, Altered microRNA profiles in bronchoalveolar lavage fluid exosomes in asthmatic patients. J Allergy Clin Immunol 2013; 131:894–903. 23333113 10.1016/j.jaci.2012.11.039 PMC4013392

[36] Francisco-Garcia AS, Garrido-Martín EM, Rupani H, Small RNA species and microRNA profiles are altered in severe asthma nanovesicles from broncho alveolar lavage and associate with impaired lung function and inflammation. Noncoding RNA 2019;5:51. 10.3390/ncrna5040051 PMC6958500 31684064

[37] Hough KP, Trevor JL, Strenkowski JG, Exosomal transfer of mitochondria from airway myeloid-derived regulatory cells to T cells. Redox Biol 2018;18:54–64. 29986209 10.1016/j.redox.2018.06.009 PMC6031096

[38] Prado N, Marazuela EG, Segura E, Exosomes from bronchoalveolar fluid of tolerized mice prevent allergic reaction. J Immunol 2008;181:1519–25. 18606707 10.4049/jimmunol.181.2.1519

[39] Torregrosa Paredes P, Esser J, Admyre C, Bronchoalveolar lavage fluid exosomes contribute to cytokine and leukotriene production in allergic asthma. Allergy 2012;67:911–9. 22620679 10.1111/j.1398-9995.2012.02835.x

[40] Tager AM, Bromley SK, Medoff BD, Leukotriene B4 receptor BLT1 mediates early effector T cell recruitment. Nat Immunol 2003;4:982–90. 12949531 10.1038/ni970

[41] Miyahara N, Ohnishi H, Matsuda H, Leukotriene B4 receptor 1 expression on dendritic cells is required for the development of Th2 responses and allergen-induced airway hyperresponsiveness. J Immunol 2008;181:1170–8. 18606670 10.4049/jimmunol.181.2.1170

[42] Draijer C, Speth JM, Penke LRK, Resident alveolar macrophage-derived vesicular SOCS3 dampens allergic airway inflammation. FASEB J 2020;34:4718–31. 32030817 10.1096/fj.201903089R PMC7433762

[43] Lässer C, O’Neil SE, Ekerljung L, Ekström K, Sjöstrand M, Lötvall J. RNA-containing exosomes in human nasal secretions. Am J Rhinol Allergy 2011;25:89–93. 21172122 10.2500/ajra.2011.25.3573

[44] Lässer C, O’Neil SE, Shelke GV, Exosomes in the nose induce immune cell trafficking and harbour an altered protein cargo in chronic airway inflammation. J Transl Med 2016;14:181. 27320496 10.1186/s12967-016-0927-4 PMC4913423

[45] Wu G, Yang G, Zhang R, Altered microRNA Expression profiles of extracellular vesicles in nasal mucus from patients with allergic rhinitis. Allergy Asthma Immunol Res 2015;7:449–57. 26122505 10.4168/aair.2015.7.5.449 PMC4509657

[46] Mills JT, Schwenzer A, Marsh EK, Airway epithelial cells generate pro-inflammatory tenascin-C and small extracellular vesicles in response to TLR3 stimuli and rhinovirus infection. Front Immunol 2019; 10:1987. 31497021 10.3389/fimmu.2019.01987 PMC6712508

[47] Sánchez-Vidaurre S, Eldh M, Larssen P, RNA-containing exosomes in induced sputum of asthmatic patients. J Allergy Clin Immunol 2017;140:1459–61.e2. 28629752 10.1016/j.jaci.2017.05.035

[48] Maes T, Cobos FA, Schleich F, Asthma inflammatory phenotypes show differential microRNA expression in sputum. J Allergy Clin Immunol 2016;137:1433–46. 27155035 10.1016/j.jaci.2016.02.018

[49] Gupta R, Radicioni G, Abdelwahab S, Intercellular communication between airway epithelial cells is mediated by exosome-like vesicles. Am J Respir Cell Mol Biol 2019;60:209–20. 30230353 10.1165/rcmb.2018-0156OC PMC6376407

[50] Kesimer M, Scull M, Brighton B, Characterization of exosome-like vesicles released from human tracheobronchial ciliated epithelium: a possible role in innate defense. FASEB J 2009;23:1858–68. 19190083 10.1096/fj.08-119131 PMC2698655

[51] Jia M, Yan X, Jiang X, Ezrin, a membrane cytoskeleton cross-linker protein, as a marker of epithelial damage in asthma. Am J Respir Crit Care Med 2019; 199:496–507. 30290132 10.1164/rccm.201802-0373OC PMC6376623

[52] Wu Q, Eickelberg O. Ezrin in asthma: a first step to early biomarkers of airway epithelial dysfunction. Am J Respir Crit Care Med 2019;199:408–10. 30383410 10.1164/rccm.201810-1964ED

[53] Park JA, Sharif AS, Tschumperlin DJ, Tissue factor-bearing exosome secretion from human mechanically stimulated bronchial epithelial cells in vitro and in vivo. J Allergy Clin Immunol 2012;130:1375–83. 22828416 10.1016/j.jaci.2012.05.031 PMC3511625

[54] Bartel S, La Grutta S, Cilluffo G, Human airway epithelial extracellular vesicle miRNA signature is altered upon asthma development. Allergy 2020;75:346–56. 31386204 10.1111/all.14008

[55] Hashimi ST, Fulcher JA, Chang MH, Gov L, Wang S, Lee B. MicroRNA profiling identifies miR-34a and miR-21 and their target genes JAG1 and WNT1 in the coordinate regulation of dendritic cell differentiation. Blood 2009;114:404–14. 19398721 10.1182/blood-2008-09-179150 PMC2927176

[56] Huang A, Yang Y, Chen S, MiR-34a promotes DCs development and inhibits their function on T cell activation by targeting WNT1. Oncotarget 2017;8:17191–201. 28199987 10.18632/oncotarget.15228 PMC5370032

[57] Wu R, Zeng J, Yuan J, MicroRNA-210 overexpression promotes psoriasis-like inflammation by inducing Th1 and Th17 cell differentiation. J Clin Invest 2018; 128:2551–68. 29757188 10.1172/JCI97426 PMC5983326

[58] Zhao C, Zhao F, Feng H, Xu S, Qin G. MicroRNA-92b inhibits epithelial-mesenchymal transition-induced migration and invasion by targeting Smad3 in nasopharyngeal cancer. Oncotarget 2017;8:91603–13. 29207670 10.18632/oncotarget.21342 PMC5710950

[59] Maroto R, Zhao Y, Jamaluddin M, Effects of storage temperature on airway exosome integrity for diagnostic and functional analyses. J Extracell Vesicles 2017;6:1359478. 28819550 10.1080/20013078.2017.1359478 PMC5556670

[60] Patente TA, Pelgrom LR, Everts B. Dendritic cells are what they eat: how their metabolism shapes T helper cell polarization. Curr Opin Immunol 2019;58:16–23. 30875606 10.1016/j.coi.2019.02.003

[61] Vallhov H, Gutzeit C, Hultenby K, Valenta R, Gronlund H, Scheynius A. Dendritic cell-derived exosomes carry the major cat allergen F el d 1 and induce an allergic immune response. Allergy 2015;70:1651–5. 26198793 10.1111/all.12701 PMC6597348

[62] Sawant DV, Wu H, Kaplan MH, Dent AL. The Bcl6 target gene microRNA-21 promotes Th2 differentiation by a T cell intrinsic pathway. Mol Immunol 2013;54:435–42. 23416424 10.1016/j.molimm.2013.01.006 PMC3602238

[63] Stritesky GL, Muthukrishnan R, Sehra S, The transcription factor STAT3 is required for T helper 2 cell development. Immunity 2011;34:39–49. 21215659 10.1016/j.immuni.2010.12.013 PMC3040244

[64] Khare A, Chakraborty K, Raundhal M, Ray P, Ray A. Cutting edge: dual function of PPARγ in CD11c+ cells ensures immune tolerance in the airways. J Immunol 2015; 195:431–5. 26062999 10.4049/jimmunol.1500474 PMC4490989

[65] Nakano H, Free ME, Whitehead GS, Pulmonary CD103(+) dendritic cells prime Th2 responses to inhaled allergens. Mucosal Immunol 2012;5:53–65. 22012243 10.1038/mi.2011.47 PMC3697034

[66] Plantinga M, Guilliams M, Vanheerswynghels M, Conventional and monocyte-derived CD11b(+) dendritic cells initiate and maintain T helper 2 cell-mediated immunity to house dust mite allergen. Immunity 2013;38:322–35. 23352232 10.1016/j.immuni.2012.10.016

[67] Furuhashi K, Suda T, Hasegawa H, Mouse lung CD103+ and CD11b high dendritic cells preferentially induce distinct CD4+ T-cell responses. Am J Respir Cell Mol Biol 2012;46:165–72. 21908266 10.1165/rcmb.2011-0070OC

[68] Raymond M, Rubio M, Fortin G, Selective control of SIRP-α-positive airway dendritic cell trafficking through CD47 is critical for the development of TH2-mediated allergic inflammation. J Allergy Clin Immunol 2009; 124:1333–42.e1. 19748659 10.1016/j.jaci.2009.07.021

[69] Rosenberg HF, Druey KM. Modeling asthma: pitfalls, promises, and the road ahead. J Leukoc Biol 2018;104:41–8. 29451705 10.1002/JLB.3MR1117-436R PMC6134392

[70] Mittelbrunn M, Gutiérrez-Vázquez C, Villarroya-Beltri C, Unidirectional transfer of microRNA-loaded exosomes from T cells to antigen-presenting cells. Nat Commun 2011;2:282. 21505438 10.1038/ncomms1285 PMC3104548

[71] Blanchard N, Lankar D, Faure F, TCR activation of human T cells induces the production of exosomes bearing the TCR/CD3/zeta complex. J Immunol 2002;168:3235–41. 11907077 10.4049/jimmunol.168.7.3235

[72] Chiou NT, Kageyama R, Ansel KM. Selective export into extracellular vesicles and function of tRNA fragments during T cell activation. Cell Rep 2018;25:3356–3370.e4. 30566862 10.1016/j.celrep.2018.11.073 PMC6392044

[73] Azoulay-Alfaguter I, Mor A. Proteomic analysis of human T cell-derived exosomes reveals differential RAS/MAPK signaling. Eur J Immunol 2018;48:1915–7. 30207595 10.1002/eji.201847655 PMC6544359

[74] Shefler I, Salamon P, Reshef T, Mor A, Mekori YA. T cell-induced mast cell activation: a role for microparticles released from activated T cells. J Immunol 2010;185:4206–12. 20810987 10.4049/jimmunol.1000409

[75] Wypych TP, Marzi R, Wu GF, Lanzavecchia A, Sallusto F. Role of B cells in T(H) cell responses in a mouse model of asthma. J Allergy Clin Immunol 2018;141:1395–410. 28889953 10.1016/j.jaci.2017.09.001 PMC6594185

[76] Admyre C, Bohle B, Johansson SM, B cell-derived exosomes can present allergen peptides and activate allergen-specific T cells to proliferate and produce TH2-like cytokines. J Allergy Clin Immunol 2007;120:1418–24. 17868797 10.1016/j.jaci.2007.06.040

[77] Hough KP, Wilson LS, Trevor JL, Unique lipid signatures of extracellular vesicles from the airways of asthmatics. Sci Rep 2018;8:10340. 29985427 10.1038/s41598-018-28655-9 PMC6037776

[78] Savina A, Furlán M, Vidal M, Colombo MI. Exosome release is regulated by a calcium-dependent mechanism in K562 cells. J Biol Chem 2003;278:20083–90. 12639953 10.1074/jbc.M301642200

[79] Trajkovic K, Hsu C, Chiantia S, Ceramide triggers budding of exosome vesicles into multivesicular endosomes. Science 2008;319:1244–7. 18309083 10.1126/science.1153124

[80] Li Y, Zheng Q, Bao C, Circular RNA is enriched and stable in exosomes: a promising biomarker for cancer diagnosis. Cell Res 2015;25:981–4. 26138677 10.1038/cr.2015.82 PMC4528056

[81] Smyth LA, Ratnasothy K, Tsang JY, CD73 expression on extracellular vesicles derived from CD4+ CD25+ Foxp3+ T cells contributes to their regulatory function. Eur J Immunol 2013;43:2430–40. 23749427 10.1002/eji.201242909

[82] Ostroukhova M, Seguin-Devaux C, Oriss TB, Tolerance induced by inhaled antigen involves CD4(+) T cells expressing membrane-bound TGF-beta and FOXP3. J Clin Invest 2004;114:28–38. 15232609 10.1172/JCI20509 PMC437966

[83] Ding FX, Liu B, Zou WJ, Li QB, Tian DY, Fu Z. Pseudomonas aeruginosa-derived exosomes ameliorates allergic reactions via inducing the Treg response in asthma. Pediatr Res 2018;84:125–33. 29795208 10.1038/s41390-018-0020-1

[84] Cañas JA, Sastre B, Mazzeo C, Exosomes from eosinophils autoregulate and promote eosinophil functions. J Leukoc Biol 2017;101:1191–9. 28096299 10.1189/jlb.3AB0516-233RR

[85] Mazzeo C, Cañas JA, Zafra MP, Exosome secretion by eosinophils: A possible role in asthma pathogenesis. J Allergy Clin Immunol 2015;135:1603–13. 25617225 10.1016/j.jaci.2014.11.026

[86] Cañas JA, Sastre B, Rodrigo-Muñoz JM, Eosinophil-derived exosomes contribute to asthma remodelling by activating structural lung cells. Clin Exp Allergy 2018;48:1173–85. 29451337 10.1111/cea.13122

[87] Moore WC, Hastie AT, Li X, Sputum neutrophil counts are associated with more severe asthma phenotypes using cluster analysis. J Allergy Clin Immunol 2014;133:1557–63. e5. 24332216 10.1016/j.jaci.2013.10.011 PMC4040309

[88] Vargas A, Roux-Dalvai F, Droit A, Lavoie JP. Neutrophil-derived exosomes: a new mechanism contributing to airway smooth muscle remodeling. Am J Respir Cell Mol Biol 2016;55:450–61. 27105177 10.1165/rcmb.2016-0033OC

[89] Majumdar R, Tavakoli Tameh A, Parent CA. Exosomes mediate LTB4 release during neutrophil chemotaxis. PLoS Biol 2016;14: e1002336. 26741884 10.1371/journal.pbio.1002336 PMC4704783

[90] Galli SJ, Nakae S, Tsai M. Mast cells in the development of adaptive immune responses. Nat Immunol 2005;6:135–42. 15662442 10.1038/ni1158

[91] Skokos D, Botros HG, Demeure C, Mast cell-derived exosomes induce phenotypic and functional maturation of dendritic cells and elicit specific immune responses in vivo. J Immunol 2003;170:3037–45. 12626558 10.4049/jimmunol.170.6.3037

[92] Skokos D, Le Panse S, Villa I, Mast cell-dependent B and T lymphocyte activation is mediated by the secretion of immunologically active exosomes. J Immunol 2001;166:868–76. 11145662 10.4049/jimmunol.166.2.868

[93] Liang Y, Qiao L, Peng X, The chemokine receptor CCR1 is identified in mast cell-derived exosomes. Am J Transl Res 2018; 10:352–67. 29511430 PMC5835801

[94] Xie G, Yang H, Peng X, Mast cell exosomes can suppress allergic reactions by binding to IgE. J Allergy Clin Immunol 2018;141:788–91. 28916187 10.1016/j.jaci.2017.07.040

[95] Lecce M, Molfetta R, Milito ND, Santoni A, Paolini R. FcεRI signaling in the modulation of allergic response: role of mast cell-derived exosomes. Int J Mol Sci 2020;21:5464. 10.3390/ijms21155464 PMC7432241 32751734

[96] Ekström K, Valadi H, Sjöstrand M, Characterization of mRNA and microRNA in human mast cell-derived exosomes and their transfer to other mast cells and blood CD34 progenitor cells. J Extracell Vesicles 2012;1:18389. 10.3402/jev.v1i0.18389 PMC3760639 24009880

[97] Yin Y, Shelke GV, Lässer C, Brismar H, Lötvall J. Extracellular vesicles from mast cells induce mesenchymal transition in airway epithelial cells. Respir Res 2020;21:1–13. 32357878 10.1186/s12931-020-01346-8 PMC7193353

[98] Ullah I, Subbarao RB, Rho GJ. Human mesenchymal stem cells-current trends and future prospective. Biosci Rep 2015;35:e00191. 25797907 10.1042/BSR20150025 PMC4413017

[99] Shahir M, Mahmoud Hashemi S, Asadirad A, Effect of mesenchymal stem cell-derived exosomes on the induction of mouse tolerogenic dendritic cells. J Cell Physiol 2020;235:7043–55. 32043593 10.1002/jcp.29601 PMC7496360

[100] Mohammadipoor A, Antebi B, Batchinsky AI, Cancio LC. Therapeutic potential of products derived from mesenchymal stem/stromal cells in pulmonary disease. Respir Res 2018; 19:218. 30413158 10.1186/s12931-018-0921-x PMC6234778

[101] Du YM, Zhuansun YX, Chen R, Lin L, Lin Y, Li JG. Mesenchymal stem cell exosomes promote immunosuppression of regulatory T cells in asthma. Exp Cell Res 2018;363:114–20. 29277503 10.1016/j.yexcr.2017.12.021

[102] Cruz FF, Borg ZD, Goodwin M, Systemic administration of human bone marrow-derived mesenchymal stromal cell extracellular vesicles ameliorates aspergillus hyphal extract-induced allergic airway inflammation in immunocompetent mice. Stem Cells Transl Med 2015;4:1302–16. 26378259 10.5966/sctm.2014-0280 PMC4622402

[103] Fang SB, Zhang HY, Wang C, Small extracellular vesicles derived from human mesenchymal stromal cells prevent group 2 innate lymphoid cell-dominant allergic airway inflammation through delivery of miR-146a-5p. J Extracell Vesicles 2020;9:1723260. 32128074 10.1080/20013078.2020.1723260 PMC7034457

[104] de Castro LL, Xisto DG, Kitoko JZ, Human adipose tissue mesenchymal stromal cells and their extracellular vesicles act differentially on lung mechanics and inflammation in experimental allergic asthma. Stem Cell Res Ther 2017;8:151. 28646903 10.1186/s13287-017-0600-8 PMC5482954

[105] Kordelas L, Rebmann V, Ludwig AK, MSC-derived exosomes: a novel tool to treat therapy-refractory graft-versus-host disease. Leukemia 2014;28:970–3. 24445866 10.1038/leu.2014.41

[106] Fu CL, Ye YL, Lee YL, Chiang BL. Effects of overexpression of IL-10, IL-12, TGF-beta and IL-4 on allergen induced change in bronchial responsiveness. Respir Res 2006;7:72. 16677403 10.1186/1465-9921-7-72 PMC1479818

[107] Reis M, Mavin E, Nicholson L, Green K, Dickinson AM, Wang XN. Mesenchymal stromal cell-derived extracellular vesicles attenuate dendritic cell maturation and function. Front Immunol 2018;9:2538. 30473695 10.3389/fimmu.2018.02538 PMC6237916

[108] Yamashita N, Tashimo H, Matsuo Y, Role of CCL21 and CCL19 in allergic inflammation in the ovalbumin-specific murine asthmatic model. J Allergy Clin Immunol 2006; 117:1040–6. 16675330 10.1016/j.jaci.2006.01.009

[109] Mattes J, Collison A, Plank M, Phipps S, Foster PS. Antagonism of microRNA-126 suppresses the effector function of TH2 cells and the development of allergic airways disease. Proc Natl Acad Sci U S A 2009;106:18704–9. 19843690 10.1073/pnas.0905063106 PMC2773983

[110] Bartel S, Carraro G, Alessandrini F, Krauss-Etschmann S, Ricciardolo FLM, Bellusci S. miR-142-3p is associated with aberrant WNT signaling during airway remodeling in asthma. Am J Physiol Lung Cell Mol Physiol 2018;315:L328–33. 29722559 10.1152/ajplung.00113.2018

[111] Roffel MP, Brandsma CA, Van Den Berge M, Unraveling the role of miR-223-3p in the regulation of airway inflammation in asthma and COPD. ERJ 2018;52:Suppl 62, PA4998.

[112] Zhuansun Y, Du Y, Huang F, MSCs exosomal miR-1470 promotes the differentiation of CD4^+^CD25^+^FOXP3^+^ Tregs in asthmatic patients by inducing the expression of P27KIP1. Int Immunopharmacol 2019;77:105981. 31685437 10.1016/j.intimp.2019.105981

[113] Tao SC, Guo SC. Extracellular vesicles: potential participants in circadian rhythm synchronization. Int J Biol Sci 2018; 14:1610–20. 30416375 10.7150/ijbs.26518 PMC6216034

[114] Kim DK, Kang B, Kim OY, EVpedia: an integrated database of high-throughput data for systemic analyses of extracellular vesicles. J Extracell Vesicles 2013;2:20384. 10.3402/jev.v2i0.20384 PMC3760654 24009897

[115] Keerthikumar S, Chisanga D, Ariyaratne D, ExoCarta: a web-based compendium of exosomal cargo. J Mol Biol 2016;428:688–92. 26434508 10.1016/j.jmb.2015.09.019 PMC4783248

[116] Pathan M, Fonseka P, Chitti SV, Vesiclepedia 2019: a compendium of RNA, proteins, lipids and metabolites in extracellular vesicles. Nucleic Acids Res 2019;47:D516–9. 30395310 10.1093/nar/gky1029 PMC6323905

[117] Arslan F, Lai RC, Smeets MB, Mesenchymal stem cell-derived exosomes increase ATP levels, decrease oxidative stress and activate PI3K/Akt pathway to enhance myocardial viability and prevent adverse remodeling after myocardial ischemia/reperfusion injury. Stem Cell Res 2013;10:301–12. 23399448 10.1016/j.scr.2013.01.002

[118] Liu L, Jin X, Hu CF, Li R, Zhou Z, Shen CX. Exosomes derived from mesenchymal stem cells rescue myocardial ischaemia/reperfusion injury by inducing cardiomyocyte autophagy via AMPK and Akt pathways. Cell Physiol Biochem 2017;43:52–68. 28848091 10.1159/000480317

[119] Zhu LL, Huang X, Yu W, Chen H, Chen Y, Dai YT. Transplantation of adipose tissue-derived stem cell-derived exosomes ameliorates erectile function in diabetic rats. Andrologia 2018;50:e12871. 10.1111/and.12871 29057541

[120] Xu B, Zhang Y, Du XF, Neurons secrete miR-132-containing exosomes to regulate brain vascular integrity. Cell Res 2017;27:882–97. 28429770 10.1038/cr.2017.62 PMC5518987

[121] Pusic AD, Kraig RP. Youth and environmental enrichment generate serum exosomes containing miR-219 that promote CNS myelination. Glia 2014;62:284–99. 24339157 10.1002/glia.22606 PMC4096126

[122] Shende VR, Goldrick MM, Ramani S, Earnest DJ. Expression and rhythmic modulation of circulating microRNAs targeting the clock gene Bmal1 in mice. PLoS One 2011;6:e22586. 21799909 10.1371/journal.pone.0022586 PMC3142187

[123] Wang C, Zhang C, Liu L, Macrophage-derived miR-155-containing exosomes suppress fibroblast proliferation and promote fibroblast inflammation during cardiac injury. Mol Ther 2017;25:192–204. 28129114 10.1016/j.ymthe.2016.09.001 PMC5363311

[124] Khalyfa A, Poroyko VA, Qiao Z, Exosomes and metabolic function in mice exposed to alternating dark-light cycles mimicking night shift work schedules. Front Physiol 2017;8:882. 29163218 10.3389/fphys.2017.00882 PMC5673652

[125] Dooner MS, Stewart C, Deng Y, Daily rhythms influence the ability of lung-derived extracellular vesicles to modulate bone marrow cell phenotype. PLoS One 2018;13:e0207444. 30475846 10.1371/journal.pone.0207444 PMC6261033

[126] Bikov A, Kunos L, Pállinger É, Diurnal variation of circulating microvesicles is associated with the severity of obstructive sleep apnoea. Sleep Breath 2017;21:595–600. 28130736 10.1007/s11325-017-1464-y

[127] Khalyfa A, Marin JM, Qiao Z, Rubio DS, Kheirandish-Gozal L, Gozal D. Plasma exosomes in OSA patients promote endothelial senescence: effect of long-term adherent continuous positive airway pressure. Sleep 2020;43:zsz217. 31552414 10.1093/sleep/zsz217 PMC7901815

[128] Koritzinsky EH, Street JM, Chari RR, Circadian variation in the release of small extracellular vesicles can be normalized by vesicle number or TSG101. Am J Physiol Renal Physiol 2019;317:F1098–110. 31390267 10.1152/ajprenal.00568.2017 PMC6879938

[129] Catalano M, O’Driscoll L. Inhibiting extracellular vesicles formation and release: a review of EV inhibitors. J Extracell Vesicles 2020;9:1703244. 32002167 10.1080/20013078.2019.1703244 PMC6968539

[130] Zhao Z, Wijerathne H, Godwin AK, Soper SA. Isolation and analysis methods of extracellular vesicles (EVs). Extracell Vesicles Circ Nucleic Acids 2021;2:80–103. 10.20517/evcna.2021.07 PMC8372011 34414401

